# Effectiveness of Cognitive Behavioral Therapy–Based Intervention in Preventing Gaming Disorder and Unspecified Internet Use Disorder in Adolescents: A Cluster Randomized Clinical Trial

**DOI:** 10.1001/jamanetworkopen.2021.48995

**Published:** 2022-02-18

**Authors:** Katajun Lindenberg, Sophie Kindt, Carolin Szász-Janocha

**Affiliations:** 1 Institute for Psychology, Goethe-University Frankfurt, Frankfurt, Germany; 2 Institute for Psychology, Heidelberg University, Heidelberg, Germany

## Abstract

**Importance:**

Behavioral addictions were introduced as novel mental disorders in the *International Classification of Diseases, 11th Revision*, based on evidence that human behavior itself can become addictive, similar to addiction to substances. However, previous studies on prevention of gaming disorder and unspecified internet use disorder lacked randomization, follow-up measurements, and diagnostic interviews that assessed incidence rates; this trial was intended to fill this gap.

**Objectives:**

To investigate whether the PROTECT (Professioneller Umgang mit technischen Medien [Professional Use of Technical Media]) intervention can reduce the symptom severity and prevent full syndrome and subthreshold onset of gaming disorder and unspecified internet use disorder in at-risk adolescents.

**Design, Setting, and Participants:**

A multicenter cluster randomized clinical trial conducted recruitment, screening, intervention delivery, and data collection among at-risk adolescents aged 12 to 18 years in 33 high schools in Germany. Inclusion criteria for the study and symptom severity analyses were elevated symptoms of gaming disorder and unspecified internet use disorder. A subsample that met the inclusion criteria for incidence analyses (no full syndrome of gaming disorder or unspecified internet use disorder, depression, or anxiety at baseline) was analyzed for illness onset. Participants were randomized to either the PROTECT intervention group or the assessment-only control group. Participants were assessed at baseline, 1-month follow-up, 4-month follow-up, and 12-month follow-up between October 1, 2015, and September 30, 2018. Based on intent-to-treat principle, data analyses were conducted from February 8, 2019, to May 7, 2021.

**Interventions:**

PROTECT, a theory-driven, manualized, cognitive behavioral therapy–based indicated preventive group intervention that is delivered in 4 sessions by trained psychologists. It targets changes in addictive reward processing and pathological cognitive mechanisms.

**Main Outcomes and Measures:**

The primary outcome was symptom severity (measured by CSAS [Computerspielabhängigkeitsskala], a modified German video game dependency scale with a score range of 0-56 [higher scores indicating greater pathology]) along with incidence rates (assessed by a structured clinical interview) after 12 months. Secondary outcomes were comorbid psychopathology and problem behaviors.

**Results:**

A total of 422 at-risk adolescents (mean [SD] age, 15.11 [2.01] years; 229 female participants [54.3%]) were randomized to either the PROTECT intervention group (n = 167; mean [SD] risk score, 29.05 [6.98]) or the assessment-only control group (n = 255; mean [SD] risk score, 26.21 [5.01]) and were included in the symptom severity analyses. Compared with the control group, the PROTECT group showed a significantly greater reduction in symptom severity of gaming disorder or unspecified internet use disorder (γ_11_ = –0.128; 95% CI, –0.246 to –0.011; *P* = .03), reflecting a 39.8% vs 27.7% reduction of symptoms with an effect size of Cohen *d* = 0.67 (baseline vs 12-month follow-up) for the PROTECT group. Differences in incidence rates did not reach statistical significance. The PROTECT group showed a significantly greater decrease in procrastination (γ_11_ = –0.458; 95% CI, –0.735 to –0.180; *P* < .001) over 12 months, but no significant differences were found for other secondary outcomes.

**Conclusions and Relevance:**

Results of this trial showed that the PROTECT intervention effectively reduced symptoms of gaming disorder and unspecified internet use disorder over 12 months. The intervention did not change incidence rates of gaming disorder or unspecified internet use disorder.

**Trial Registration:**

ClinicalTrials.gov Identifier: NCT02907658

## Key Points

**Question:**

Is manualized cognitive behavioral therapy–based indicated prevention effective in reducing symptoms of gaming disorder and unspecified internet use disorder and rates of these disorders in at-risk high school students?

**Findings:**

In this cluster randomized clinical trial of 422 at-risk adolescents with gaming disorder and unspecified internet use disorder, the PROTECT (Professioneller Umgang mit technischen Medien [Professional Use of Technical Media]) intervention group had a significantly greater reduction in symptoms over 12 months compared with the assessment-only control group (39.8% vs 27.7%). Differences in incidence rates did not reach significance.

**Meaning:**

Findings of this trial indicate that the PROTECT intervention in high schools is effective in reducing symptoms of gaming disorder and unspecified internet use disorder.

## Teaser

This cluster randomized clinical trial evaluates the effect of a cognitive behavioral therapy–based intervention on symptom severity, incidence rate, and psychopathology and behaviors associated with gaming disorder and unspecified internet use disorder among high school students in Germany.

## Introduction

With the release of the *International Classification of Diseases, 11th Revision (ICD-11)*, the World Health Organization recognized that excessive behaviors can become addictive, and such an addiction can be analogous to addictions based on psychotropic substances.^1^ Because of the neurobiological similarity between substance-related and nonsubstance-related addictions,^2,3,4,5,6^ these disorders were nosologically classified as “disorders due to substance use or addictive behaviors.”^1^ Gaming disorder (*ICD-11* code 6C51) was included in *ICD-11* as a novel behavioral addiction in addition to gambling disorder, which was listed as an impulse control disorder in earlier editions of the *ICD*. Other behavioral addictions, such as internet use disorder, were recommended for inclusion as “other specified” (*ICD-11* code 6C5Y) or “unspecified” (*ICD-11* code 6C5Z) disorders that are attributed to addictive behaviors.^7^ Scientists have called for intensive research on prevention of (internet) gaming disorder.^8^

Gaming disorder and unspecified internet use disorder are associated with numerous impairments, such as comorbid psychiatric disorders, lower life satisfaction, and lower academic achievements.^9,10,11,12,13,14,15,16,17,18,19,20,21,22^ Epidemiologic studies show a relevant prevalence of gaming disorder (4.6%)^23^ and internet use disorder (6.0%),^24^ including both gaming disorder and unspecified internet use disorder. Adolescents seem to be particularly vulnerable to developing disorders that are associated with the reward system.^20,25,26,27^ In line with this finding, excessive use of video games and the internet is highly prevalent in youth and early adulthood.^23,28,29,30^ In 1 study, prevalence of gaming disorder and unspecified internet use disorder increased from 2.8% in children aged 11 to 12 years to 9.1% in young adults aged 18 to 21 years,^28^ whereas several studies found that prevalence decreased at the end of the third decade of life.^20,25,27^ Findings on the stability of addiction symptoms over 1 year have been mixed, ranging from 28.4%^31^ to 37.6%^32^ to 63.3%.^33^ However, those patients whose addictive behaviors persist present a challenge to health care and social systems. Individuals with these behaviors show limited motivation to seek help and treatment,^34,35^ which emphasizes the need to prevent illness onset. The excessive use of video games and internet applications has been growing (particularly during the ongoing COVID-19 pandemic),^29,36,37^ which underlines the need for prevention and early intervention.^38,39,40,41,42,43^ Between September 2019 and March 2020, the mean amount of time that adolescents in Germany spent on video gaming increased by 75.0% on weekdays (Monday through Friday) and by 29.3% on weekends.^29^

The American Psychological Association recommendations for efficient psychological prevention emphasize a theoretical foundation for the intervention, an optimal dose-response relationship, and systemic anchoring (eg, in schools).^44^ Typically, prevention should start before symptom manifestation and should target individuals who might gain the most benefit and are selected according to factors that increase the risk of illness onset, such as age and first symptoms (ie, selective-indicated prevention). Risk selection potentially enhances cost-effectiveness. Prevention programs that target at-risk individuals must demonstrate incremental effectiveness beyond the expected effects of spontaneous remission and regression to the mean. Therefore, it is of utmost necessity to design longitudinal, randomized clinical efficacy trials that allow the observation of natural symptom courses in a control group and that use clinically relevant end points (ie, reduction of first symptoms and prevention of illness onset). The quality of previous studies on prevention of gaming disorder and unspecified internet use disorder has often been criticized because they lacked randomization as well as follow-up measurements and diagnostic interviews that assessed incidence rates.^8,45,46^

To address this significant gap, we conducted a 2-group, cluster randomized clinical trial of the long-term effects of the PROTECT (Professioneller Umgang mit technischen Medien [Professional Use of Technical Media])^47^ intervention (eFigure 1 in Supplement 1) for indicated prevention of gaming disorder and unspecified internet use disorder, which follows the American Psychological Association guidelines for prevention in psychology. We investigated whether the PROTECT intervention can reduce the symptom severity and prevent full syndrome and subthreshold onset of gaming disorder and unspecified internet use disorder in at-risk adolescents.

## Methods

The data presented herein were obtained from the preregistered PROTECT study. The trial protocol (Supplement 2) was approved by the University of Education Heidelberg Research Ethics Committee and the Regional Council. All high schools in the Rhine-Neckar metropolitan region in Germany were contacted via the headmaster’s office, and 33 high schools participated on a voluntary basis. Written informed consent was obtained from all participants. Data were collected between October 1, 2015, and September 30, 2018, and data were prepared, coded, and analyzed through May 7, 2021. We used the Consolidated Standards of Reporting Trials (CONSORT) reporting guideline.

### Screening of At-Risk Participants and Randomization

Participants were screened for risk before study enrollment using the German version of the Compulsive Internet Use Scale (CIUS).^48^ A CIUS score of 24 or higher, which is commonly used to identify high-risk participants, has been found to identify cases with a sensitivity of at least 70%.^49^ To increase sensitivity in the present study but also limit the total number needed to treat, we chose a CIUS score of 20 as the cutoff criterion and thus included participants at moderate risk and high risk in the study. This at-risk subsample, which was eligible to participate, included the upper 36.4% of all screened participants. The internal consistency at screening was high (Cronbach α = .87).

**Figure 1.**
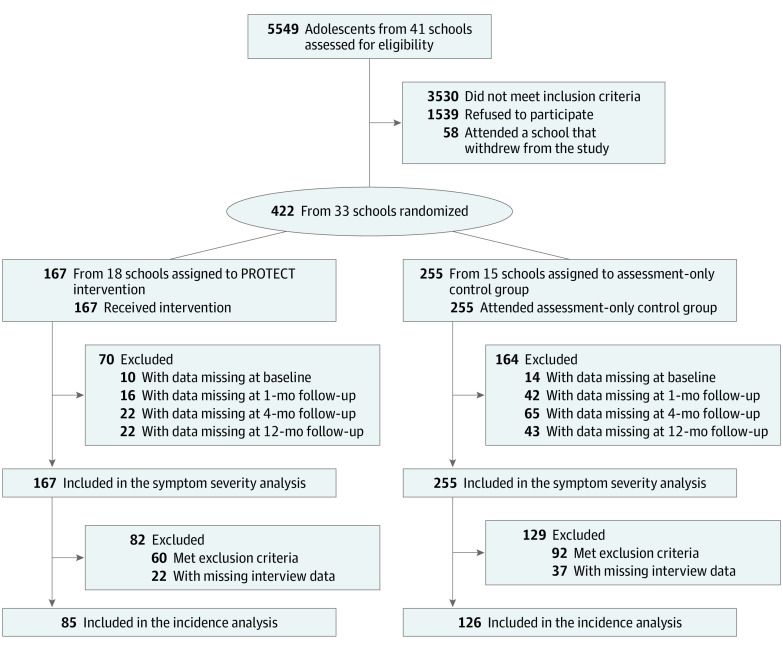
CONSORT Diagram of Participant Flow Through Trial The first primary end point was symptom severity as analyzed by 3-level hierarchical growth curve models, allowing for missing data. The second primary end point was 12-months’ incidence rate, which required narrower inclusion criteria and clinical interview.

We screened 5549 high school students aged 12 to 18 years for risk of gaming disorder and unspecified internet use disorder before enrollment and randomization to either the PROTECT intervention group (n = 167) or the assessment-only control group (n = 255) (Figure 1). Randomization was conducted in schools, which were stratified by academic level (low, medium, or high), as clusters by an independent person who used MATLAB (MathWorks) to generate the 3 randomization lists (each with permuting block randomization, with block sizes of 4-6). The refusal or agreement to participate was recorded before the schools were randomized. Descriptive statistics by group are presented in eTable 1 in Supplement 1.

All participants were assessed (by paper and pencil) at baseline, 1-month follow-up, 4-month follow-up, and 12-month follow-up and were included in the symptom severity analyses. Following the trial protocol (Supplement 2), we tested for illness onset (clinical interview) at 12-month follow-up and included in the incidence analyses a subsample of 211 eligible participants (85 from the PROTECT intervention group, and 126 from the assessment-only control group). Participants in the subsample had no clinically relevant gaming disorder or unspecified internet use disorder and met 5 or more diagnostic criteria on the CSAS [Computerspielabhängigkeitsskala], a modified German video game dependency scale; had no depression (DIKJ [Depressions-Inventar für Kinder und Jugendliche] questionnaire T score ≥60); and had no social anxiety (Social Interaction Anxiety Scale total score ≥36) at baseline. The flow of participants is presented in Figure 1. Detailed information on the participant base for the incidence analyses is presented in eFigure 2 in Supplement 1.

### Treatment, Assessment, and Blinding

PROTECT is a theory-driven, school-based, manualized, cognitive behavioral therapy (CBT)–based indicated preventive group intervention. It consists of four 90-minute sessions and is delivered by 2 trained psychologists per group.^47^ Previous research found the best evidence for CBT-based programs for treatment and early intervention for gaming disorder and unspecified internet use disorder.^35,50,51,52,53^

Risk screenings, paper-and-pencil assessments, the diagnostic interview, and the PROTECT intervention delivery were conducted during regular school hours by trained psychologists. The structured clinical interview was recorded on audiotape. Recordings were coded by a second, blinded rater.

We also assessed sex, age, school type, grades, sick days within the past month, and mean time spent online. Race and ethnicity data were not collected.

### Primary and Secondary End Points

The primary end point was the symptom severity of gaming disorder or unspecified internet use disorder, as assessed by the CSAS (score range: 0-56, with higher scores indicating greater pathology).^14^ With permission from the CSAS publisher, we adapted the CSAS items to cover both gaming disorder and unspecified internet use disorder in a common score (eg, item 1: “Even when I am not gaming/online, I think about online gaming/the Internet” for preoccupation). In addition, using the structured clinical interview, we assessed incidence rates of full-syndrome gaming disorder or unspecified internet use disorder (defined as meeting ≥5 diagnostic criteria of the *Diagnostic and Statistical Manual of Mental Disorders,* Fifth Edition [*DSM-5*]) and subthreshold gaming disorder or unspecified internet use disorder (defined as meeting ≥3 diagnostic criteria of the *DSM-5*). The clinical interview covered the 9 *DSM-5* criteria for internet gaming disorder that were adapted for gaming disorder and unspecified internet use disorder separately, following a branched structure of 107 structured questions per section (214 questions in total).

The secondary end points were procrastination, general psychopathology, depressive symptoms, social anxiety, performance anxiety and school anxiety, emotion regulation, school-related social behavior and learning behavior, and self-efficacy. These comorbid psychopathology and problem behaviors have been found to be associated with gaming disorder and unspecified internet use disorder.^9,10,11,12,13,16,18,19,20,21,22,25,54,55,56,57,58,59,60,61,62,63,64,65^ Detailed descriptions of all outcome measures are provided in the eAppendix in Supplement 1 and in the trial protocol in Supplement 2.

### Statistical Analysis

**Figure 2.**
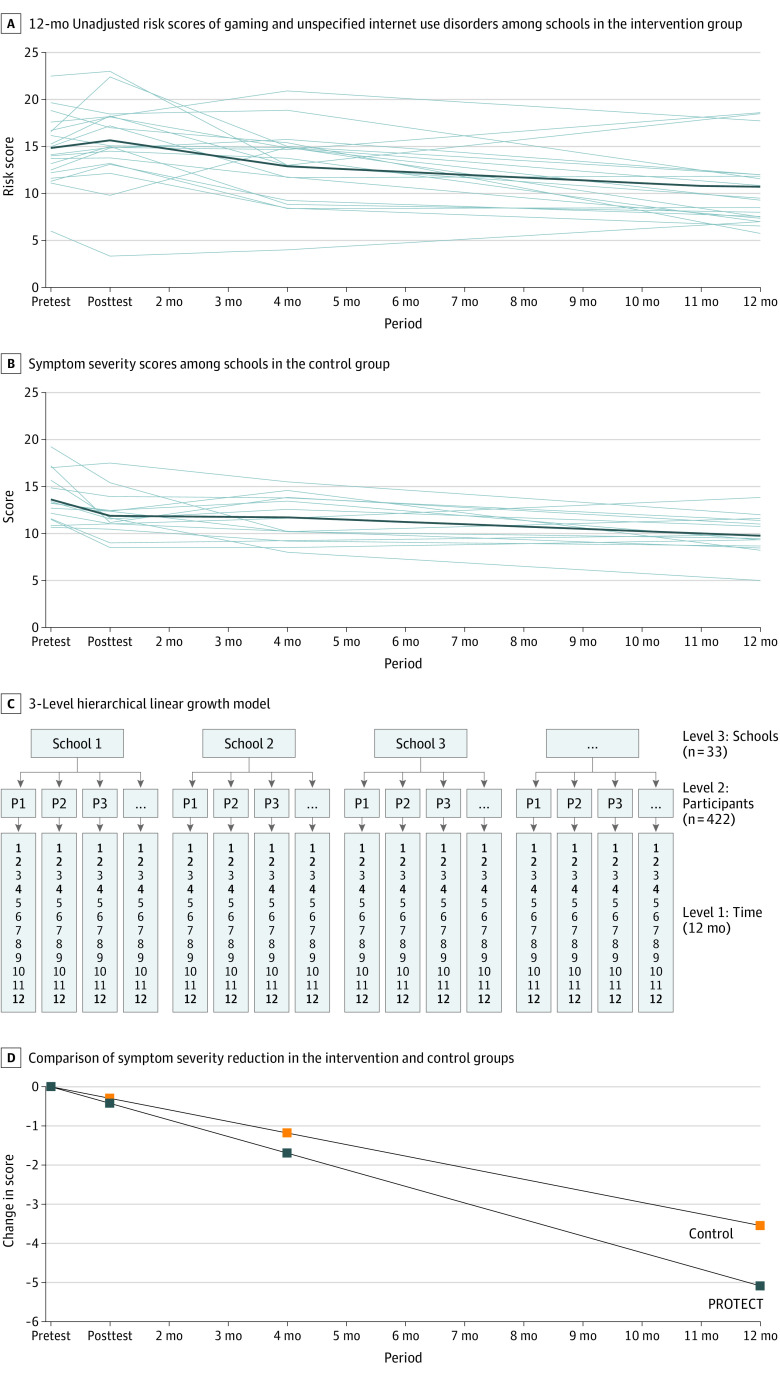
Gaming Disorder or Internet Use Disorder Symptom Changes Over 12 Months A-B, Each line represents the mean scores of 1 high school. PROTECT indicates Professioneller Umgang mit technischen Medien (Professional Use of Technical Media).

According to a previous sample size calculation,^66^ a total number of 340 participants (170 per group) was needed to ensure a power of 80%, and a 2-sided α = .05 was needed to detect an effect that would reduce incidence rates by one-third through the intervention (incidence rate of 36% [36 per 100 participants] instead of 24% [24 per 100 participants]). To analyze symptom severity (continuous variable), a 3-level hierarchical linear growth curve model was used as the statistical method, which allowed us to model change in nested data in a repeated-measurement design (level 1 units indicating time, level 2 units indicating participants, and level 3 units indicating schools) (Figure 2C). Significant baseline differences were considered by including level-2 and level-3 random intercepts. The rate of change (the slope of the curve) was estimated by the interaction between the time and PROTECT parameters (γ_11_). The time parameter was scaled from 0 to 12, representing 1 unit per month, and the PROTECT parameter was dummy coded (1 for the PROTECT intervention group, and 0 for the assessment-only control group). A more detailed description of the 3-level hierarchical linear growth curve model specification is provided in the eAppendix in Supplement 1.

To compare incidence rates between groups, we used χ^2^ statistic to analyze the number of individuals who had full-syndrome gaming disorder or unspecified internet use disorder (met ≥5 *DSM-5* criteria) vs those who had subthreshold gaming disorder or unspecified internet use disorder (met ≥3 *DSM-5* criteria). Because of an unbalanced risk of illness onset between groups at baseline (moderate risk [CIUS score between 20 and 23] vs high risk [CIUS score ≥24]: PROTECT intervention group, 29.4% vs 70.6%; assessment-only control group: 41.3% vs 58.7%), we analyzed incidence rates stratified by risk score.

All significance tests were 2-sided, and *P* < .05 was used to indicate significance. All statistical calculations used IBM SPSS Statistics, version 27 (IBM). Based on intent-to-treat principle, data analyses were conducted from February 8, 2019, to May 7, 2021.

## Results

A total of 422 at-risk adolescents (mean [SD] age, 15.11 [2.01] years; 229 female [54.3%] and 193 male [45.7%] participants) were randomized to the PROTECT intervention group (n = 167; mean [SD] risk score, 29.05 [6.98]) or to the assessment-only control group (n = 255; mean [SD] risk score, 26.21 [5.01]) and were included in the symptom severity analyses.

The intervention was delivered in 24 groups consisting of 3 to 11 adolescents. The mean (SD) number of attended sessions was 3.7 (0.45) of 4 sessions. Participants evaluated the intervention favorably: 90.7% (n = 137) were satisfied with PROTECT, and 85.5% (n = 127) would recommend PROTECT to a friend. The mean evaluation score was 7.53 out of 10 points, with 1 being very poor and 10 being very good.

### Primary Outcomes

Raw scores of the symptom courses of gaming disorder and unspecified internet use disorder over 12 months, stratified by groups and schools, are displayed in Figure 2A and B. The raw scores showed an initial increase in symptom severity in the PROTECT intervention group (Figure 2A) within the first month, compared with a decrease in symptom severity in the assessment-only control group (Figure 2B), followed by a larger decrease in symptom severity in the PROTECT intervention group between months 2 and 12. Descriptive statistics and effect sizes of gaming disorder and unspecified internet use disorder symptom courses stratified by group are shown in eTable 2 in Supplement 1. Group means were corrected by level-3 baseline differences. Level-3 baseline data are presented in eTable 3 in Supplement 1.

We found a significantly greater reduction in symptom severity of gaming disorder and unspecified internet use disorder in the PROTECT intervention group compared with the assessment-only control group (γ_11_ = –0.128; 95% CI, –0.246 to –0.011; *P* = .03) as demonstrated by a significantly steeper slope in Figure 2D. The incremental improvement among PROTECT participants compared with control participants represented a 39.8% vs 27.7% reduction of symptoms over 12 months, with an effect size of Cohen *d* = 0.67 in the PROTECT group. Parameter estimates of fixed effects are shown in the Table. Random effects and a comparison of model fit are shown in eTable 4 in Supplement 1.

**Table.** Results of Fixed-Effects Parameters for Symptom Severity

Variable	Parameter^a^	Estimate	SE	*t* value	*P* value	(95% CI)
Gaming disorder or unspecified internet use disorder symptom severity, assessed by CSAS	Intercept (γ_00_)	12.762	0.565	22.592	<.001	(11.589 to 13.934)
	Time (γ_10_)	–0.295	0.038	–7.775	<.001	(–0.379 to 0.221)
	PROTECT × Time (γ_11_)	–0.128	0.060	–2.148	.03	(–0.246 to –0.011)

Abbreviations: CSAS, Computerspielabhängigkeitsskala (modified German video game dependency scale; score range: 0-56, with higher scores indicating greater pathology); PROTECT, Professioneller Umgang mit technischen Medien) Professional Use of Technical Media).

^a^ The time parameter was scaled from 0 to 12, representing 1 unit per month. The PROTECT parameter was dummy coded, with 1 for the PROTECT intervention group and 0 for the assessment-only control group.

A total of 12 participants (5.7%) developed unspecified internet use disorder (6 in the PROTECT group and 6 in the control group), meeting at least 5 *DSM-5* diagnostic criteria of internet gaming disorder after 12 months. Any subthreshold gaming disorder (n = 10 [3 in the PROTECT group and 7 in the control group]) or unspecified internet use disorder (n = 33 [10 in the PROTECT group and 23 in the control group]) that met 3 or 4 *DSM-5* diagnostic criteria of internet gaming disorder was found in 40 individuals (19.0% of the sample). Three participants (1.4%) met *DSM-5* diagnostic criteria for both subthreshold gaming disorder and subthreshold unspecified internet use disorder. Differences in incidence rates between treatment groups were not significant. In the high-risk group, incidence rates for subthreshold gaming disorder or unspecified internet use disorder were 18.3% (n = 11) in the PROTECT intervention group and 29.7% (n = 22) in the assessment-only control group (χ^2^ = 0.420; *P* = .09) (eTable 5 in Supplement 1).

### Secondary Outcomes

Pearson correlations of secondary outcomes with gaming disorder or unspecified internet use disorder are presented in eTable 6 in Supplement 1. Group differences in secondary outcomes were analyzed by comparing the slopes (interaction between time and group) in 3-level hierarchical linear growth models (random intercept and random slope). We found a significantly greater reduction in procrastination in the PROTECT intervention group compared with the assessment-only control group (γ_11_ = –0.458; 95% CI, –0.735 to –0.180; *P* < .001) (eFigure 3 in Supplement 1). Fixed and random effects for procrastination and a comparison of model fit are shown in eTable 7 in Supplement 1.

Over time, the secondary outcome measures of general psychopathology, depressive symptoms, social anxiety, emotion regulation, and school-related social and learning behaviors showed significant improvement in both groups. Yet the interaction between time and group did not reach significance. Other secondary outcomes did not differ significantly between groups. Descriptive statistics and effect sizes of secondary outcomes are presented in eTable 8 in Supplement 1, and parameter estimates of fixed effects can be found in eTable 9 in Supplement 1. Because of multiple comparisons, the α level was corrected by the number of tests using the Bonferroni correction (α = .05 divided by 9 = .006).

## Discussion

To our knowledge, this trial is the first to investigate the long-term effects of a school- and CBT-based indicated preventive intervention (PROTECT) for symptom reduction of gaming disorder or unspecified internet use disorder in adolescents vs an assessment-only control group. We believe it is also the first study in the field to be preregistered and to use a theory-driven, manualized intervention in accordance with American Psychological Association guidelines,^44^ and to analyze incidence rates as measured by a diagnostic interview. The findings from this trial correspond with previous findings on psychotherapeutic treatment of gaming disorder and unspecified internet use disorder, which demonstrated the beneficial effects of CBT-based interventions on symptom severity.^35,50,51,52,53^

Results indicated a significantly greater reduction in symptom severity of gaming disorder or unspecified internet use disorder in the PROTECT intervention group compared with the assessment-only control group. Although both groups showed a significant symptom reduction over 12 months, a significantly greater incremental effect was found in the PROTECT intervention group. This finding indicates that the intervention had an effect that was above and beyond spontaneous remission. To our knowledge, only 1 other prevention study with a randomized clinical design could prove preventive effects.^67^ In contrast to the PROTECT intervention, the other preventive approach was a universal, knowledge-based, media-literacy curriculum that addressed unselected adolescents in 6th and 7th grades between 2010 and 2012.^67^

Incidence rates were lower than expected. The number of subthreshold cases that we found was approximately equal to the number of expected full-syndrome cases. Descriptive analyses showed that in individuals with high risk at baseline, fewer participants in the PROTECT intervention group than in the assessment-only control group developed a full-syndrome or subthreshold gaming disorder or unspecified internet use disorder. However, the power was too low to statistically validate the effect, and the study did not find a significant reduction in incidence rates. Power analysis was sensitive to base rate overestimation or underestimation, and the incidence rate that was identified by clinical interviews in this at-risk population was much lower than assumed based on paper and pencil–based epidemiologic studies (trial protocol in Supplement 2).^66^ Nevertheless, we believe incidence rates that were assessed by structured clinical interviews should be considered as the ultimate proof of preventive effects and should be an approach used in future studies. To avoid underpowered samples, adaptive designs that allow for a sample size recalculation after a planned interim analysis could be a method of choice when exact base rate estimations are unknown.

The spontaneous symptom reduction effect on gaming disorder or unspecified internet use disorder in the control group was higher than expected. This finding is in line with results from studies that indicated a rather low temporal symptom stability in adolescents and high spontaneous remission rates over 1 year.^31,32^ However, it could also be a regression to the mean effect or an increased problem awareness. Moreover, the symptom reduction in the PROTECT intervention group was significantly greater than that in the assessment-only control group, suggesting a true effect of the PROTECT intervention that went beyond mere problem awareness, regression to the mean, or spontaneous remission.

In addition, descriptive symptom analyses showed an initial increase in symptom severity of gaming disorder or unspecified internet use disorder within the first month in the PROTECT intervention group, compared with a decrease in symptom severity in the assessment-only control group. A similar result was found in another study that assessed the effects of an early intervention program (called PROTECT+), which was developed and conducted at our university and was based on the same concept.^50^ This paradox reaction could be explained by an elevated awareness of problematic internet behavior, which was induced by the PROTECT intervention. It seemed unlikely that the intervention itself was harmful because symptoms significantly decreased at the 4-month follow-up and the 12-month follow-up.

Besides the effects of the PROTECT intervention on the primary outcome, we found significant incremental effects on procrastination as a secondary outcome. Previous research has shown that procrastination is closely related to gaming disorder and unspecified internet use disorder.^65,68,69^ The specificity of the intervention’s effects on gaming disorder, unspecified internet use disorder, and procrastination symptoms vs other comorbid symptoms might be explained by the content of the PROTECT intervention manual, which specifically addresses 3 problem behaviors: (1) boredom and motivational problems, (2) procrastination and test anxiety, and (3) social anxiety. Although procrastination was decreased significantly, the effects of the interaction between time and group on social anxiety as well as on school and performance anxiety were marginally significant, which is a promising result and a step in the right direction.

Other secondary outcome measures (general psychopathology, depressive symptoms, social anxiety, emotion regulation, and school-related social and learning behaviors) improved in both groups over time. Yet the interaction between time and group did not reach significance. All secondary outcomes were associated with gaming disorder and unspecified internet use disorder (small to medium-size effects; correlations are presented in eTable 6 in Supplement 1). Thus, a decrease in comorbid symptoms along with a decrease in symptoms of gaming disorder or unspecified internet use disorder were consistent with our assumptions. The dose (four 90-minute sessions) was not high enough to achieve statistically significant effects on all comorbid symptoms, which were more generic and not directly addressed by the PROTECT intervention.

Prevention of gaming disorder or unspecified internet use disorder is especially relevant in the ongoing COVID-19 pandemic.^29,36,37^ The knowledge gained from this trial may be applied in follow-up studies using larger samples and focusing on high-risk participants to confirm a reduction in incidence rates. In addition, further investigation into the effectiveness of the PROTECT intervention in a routine setting is needed, in which educators instead of trained psychologists deliver the intervention.

### Limitations

This study has several limitations. First, the proportion of eligible adolescents who participated in the study was only 1 of 5. This proportion might limit the generalizability of the findings; however, we did not find systematic differences in screening data between adolescents who agreed to participate and those who refused to participate. Moreover, this proportion is in line with previous research that demonstrated low help-seeking behavior and treatment motivation associated with gaming disorder and unspecified internet use disorder.^34,35,70^ Second, the number of incidence events was lower than expected, leading to an underpowered sample for the incidence analyses. Third, because of limited resources, we conducted diagnostic clinical interviews only at the 12-month follow-up to assess incidence rates, and we used questionnaire data to exclude cases that met 5 or more *DSM-5* criteria at baseline. Fourth, we found differences in all outcome measures between schools, which were reflected in the disparities between the treatment conditions because of cluster randomization. These differences were controlled for in all statistical analyses. Yet these variations cannot be explained by differences in educational level^69^ or by any other variable that we assessed, and the reason for the differences between schools remains open to speculation. We recommend the use of randomization within schools (individuals within schools as the unit) in future studies, although this approach might be logistically more challenging.

## Conclusions

To our knowledge, this cluster randomized clinical trial is the first to investigate the long-term effects of a manualized prevention program (PROTECT). This intervention effectively reduced symptoms of gaming disorder or unspecified internet use disorder over 12 months, which is a clinically, scientifically, and politically important step in dealing with this newly recognized disorder. Knowledge gained from this trial could be used in follow-up studies with larger samples and high-risk participants to confirm the reduction in incidence rates. Further research is needed to investigate the effectiveness of the PROTECT intervention in a routine setting in which educators deliver the intervention.

## Notes

Supplement 1. **eAppendix.** Descriptions of Procedure, Outcomes, and Model Specification **eFigure 1.** The PROTECT Intervention for the Prevention of Gaming Disorder and Unspecified Internet Use Disorder **eFigure 2.** Flow of Participants for Incidence Analysis **eFigure 3.** Procrastination Symptom Changes Over 12 Months **eTable 1.** Descriptive Statistics Separated by Group **eTable 2.** Descriptive Statistics and Effect Sizes of Primary Outcomes **eTable 3.** Level 3 Baseline Data (Means) by School **eTable 4.** Parameter Estimates for Multilevel Linear Growth Model Examining GD/Unspecified IUD Symptom Reduction **eTable 5.** 12-Months Incidence Rates by Group, Stratified by Baseline Risk of Illness-Onset **eTable 6.** Correlation Matrix of GD/Unspecified IUD Symptoms With Comorbid Symptoms at Baseline **eTable 7.** Parameter Estimates for Multilevel Linear Growth Model Examining Procrastination Symptom Reduction **eTable 8.** Descriptive Statistics and Effect Sizes of Secondary Outcomes **eTable 9.** Results of Fixed Effects Parameters for Secondary Outcome Measures
eReferences

Supplement 2.
Trial Protocol

Supplement 3.
Data Sharing Statement
