# Supplementary material for: Effectiveness of Cognitive Behavioral Therapy–Based Intervention in Preventing Gaming Disorder and Unspecified Internet Use Disorder in Adolescents: A Cluster Randomized Clinical Trial
Source: JAMA Netw Open. 2022 Feb 18;5(2):e2148995. doi: 10.1001/jamanetworkopen.2021.48995 (PMC8857686; doi:10.1001/jamanetworkopen.2021.48995)
Supplement: Supplement 1. — eAppendix. Descriptions of Procedure, Outcomes, and Model Specification eFigure 1. The PROTECT Intervention for the Prevention of Gaming Disorder and Unspecified Internet Use Disorder eFigure 2. Flow of Participants for Incidence Analysis eFigure 3. Procrastination Symptom Changes Over 12 Months eTable 1. Descriptive Statistics Separated by Group eTable 2. Descriptive Statistics and Effect Sizes of Primary Outcomes eTable 3. Level 3 Baseline Data (Means) by School eTable 4. Parameter Estimates for Multilevel Linear Growth Model Examining GD/Unspecified IUD Symptom Reduction eTable 5. 12-Months Incidence Rates by Group, Stratified by Baseline Risk of Illness-Onset eTable 6. Correlation Matrix of GD/Unspecified IUD Symptoms With Comorbid Symptoms at Baseline eTable 7. Parameter Estimates for Multilevel Linear Growth Model Examining Procrastination Symptom Reduction eTable 8. Descriptive Statistics and Effect Sizes of Secondary Outcomes eTable 9. Results of Fixed Effects Parameters for Secondary Outcome Measures eReferences [file jamanetwopen-e2148995-s001.pdf]

Supplementary Online Content

Lindenberg K, Kindt S, Szász-Janocha C. Effectiveness of cognitive behavioral therapy–based intervention in preventing gaming disorder and unspecified internet use disorder in adolescents: a cluster randomized clinical trial. *JAMA Netw Open*. 2022;5(2):e2148995. doi:10.1001/jamanetworkopen.2021.48995

eAppendix. Descriptions of Procedure, Outcomes, and Model Specification
eFigure 1. The PROTECT Intervention for the Prevention of Gaming Disorder and Unspecified Internet Use Disorder
eFigure 2. Flow of Participants for Incidence Analysis
eFigure 3. Procrastination Symptom Changes Over 12 Months
eTable 1. Descriptive Statistics Separated by Group
eTable 2. Descriptive Statistics and Effect Sizes of Primary Outcomes
eTable 3. Level 3 Baseline Data (Means) by School
eTable 4. Parameter Estimates for Multilevel Linear Growth Model Examining GD/Unspecified IUD Symptom Reduction
eTable 5. 12-Months Incidence Rates by Group, Stratified by Baseline Risk of Illness-Onset
eTable 6. Correlation Matrix of GD/Unspecified IUD Symptoms With Comorbid Symptoms at Baseline
eTable 7. Parameter Estimates for Multilevel Linear Growth Model Examining Procrastination Symptom Reduction
eTable 8. Descriptive Statistics and Effect Sizes of Secondary Outcomes
eTable 9. Results of Fixed Effects Parameters for Secondary Outcome Measures
eReferences

This supplementary material has been provided by the authors to give readers additional information about their work.

© 2022 Lindenberg K et al. *JAMA Network Open.*

eAppendix. Descriptions of Procedure, Outcomes, and Model Specification

Description of Procedure

The presented data base upon the pre-registered PROTECT study (ClinicalTrials.gov: NCT02907658), which was funded by the Dietmar Hopp Foundation. The published study protocol[1] (Supplement 1) was approved by the University of Education Heidelberg Research Ethics Committee on September 3, 2015 (Az.: 7741.35-13). Approval from the Regional Council was obtained on October 19, 2015 (Az.: 71c2-6499.25).

Participants were recruited from October 2015 to December 2016 in 41 interested high-schools, of which 33 finally participated. All high-schools in the Rhine-Neckar metropolitan region were contacted via the headmaster's office and participated on a voluntary basis. Eligible students and their parents received detailed information about the relevance, aims, and procedure of the study. Informed written consent was obtained from all participants and from their legal guardians. The PROTECT program as well as the assessments were conducted in situ at the schools between October 2015 and September 2018.

Description of Outcomes

Time spent online was assessed separately for weekdays (Monday through Friday) and weekends. The total average time per day was computed as follows: *(5*average time on weekdays + 2*average time on weekends)/7.* In addition, we assessed the frequency of gaming, chatting and surfing.

We adapted the CSAS items to cover both GD and unspecified IUD (e.g., item 1: "Even when I am not gaming/online, I think about online gaming/the Internet" for preoccupation) with permission by the publisher. Additionally, we assessed *incidence rates of GD and unspecified IUD*. The CSAS includes all 9 diagnostic criteria for Internet Gaming Disorder as defined in the DSM-5. These criteria are assessed by 2 items each (18 in total) on a 4-point Likert scale from 0 to 3 ("strongly disagree", "somewhat disagree", "somewhat agree", "strongly agree"), resulting in a range of 0-56. A criterion of IGD is met, if at least one of the items has been rated with 3 ("strongly agree").

© 2022 Lindenberg K et al. *JAMA Network Open.*

Participants in both arms of the study were assessed at the 12-month follow-up using a clinical interview based on the criteria of IGD as proposed in the DSM-5. To assess both gaming and non-gaming subtypes, the interview includes two separate sections to assess GD and unspecified IUD subsequently. It contains 107 structured questions per section (214 in total), assessing the following nine criteria for GD and unspecified IUD according to a branched structure. Full-syndrome GD or unspecified IUD was defined by meeting 5 or more criteria. Subthreshold GD or unspecified IUD was defined by meeting 3 or more criteria.

Procrastination was assessed with the German Procrastination Questionnaire (APROF)[2]. *General psychopathology* was assessed with the Strengths and Difficulties Questionnaire (SDQ)[3]. We assessed symptoms of *depression* using the German Depression Inventory for Children and Adolescents (DIKJ)[4,5]. *Social anxiety* was measured with the German version of the Social Interaction Anxiety Scale (SIAS)[6,7]. We assessed *performance anxiety and school anxiety* using a subscale of the German revision of the Fear Survey Schedule for Children (PHOKI)[8,9]. To assess *emotion regulation strategies*, we used the German Questionnaire for the Assessment of Emotion Regulation in Children and Adolescents (FEEL-KJ)[10]. We used the fear and sadness items (60), which can be rated on a 5-point scale from 1 ("almost never") to 5 ("almost always") and calculated the scores of *adaptive emotion regulation strategies* and *maladaptive emotion regulation strategies* across emotions. *Social behavior and learning behavior* was assessed with the German Student Assessment List for Social and Learning Behavior (SSL)[11]. We assessed *self-efficacy* using the German General Self-Efficacy Scale (SWE)[12]. Adverse events were not recorded.
The assessments and interviews took place in the schools, so the interviewer or people who administered the assessments could not be blinded. However, the audiotapes were recorded anonymously so that the second rater could be blinded.

Description of Model Specification

We included participants with missing data in the analysis, because baseline data did not differ significantly between participants who were lost to follow-up and participants with complete datasets. Missing values were not imputed. Prior to specifying models, all outcome data were tested for statistical assumptions. Variables were fitted to the 3-level hierarchical linear growth models and tested in three steps. In step one, we nested variance components

© 2022 Lindenberg K et al. *JAMA Network Open.*

in 3 levels, i.e., time within individuals within schools, and computed an unconditional means model (model 0), describing outcome variation as a function of initial status. Thus, we included initial status as a fixed effect parameter (intercept $\gamma 00$) to predict the outcome. Residual variance components were used to analyze significant systematical variation, justifying further model specification. Significant residual variance components at level 1 indicate systematic variation left within-persons (σ^2_ε) and between-persons (σ^2_0) and justify the inclusion of additional parameters that might explain intra-individual differences (i.e., symptom change over time) and inter-individual differences (i.e., group differences). Thus, in step two, we specified unconditional growth models (model 1), including the rate of change (slope γ_{10}) as additional fixed effect parameter to initial status (intercept γ_{00}) to predict the outcome. In step three (model 2), we specified conditional growth models with random intercepts and random slopes, including the group parameter (γ_{11}; coded as PROTECT=1) as additional predictor for the outcome. Models were compared by fit parameters (AIC and -2 log-likelihood). All analyses were conducted using IBM SPSS 27.

© 2022 Lindenberg K et al. *JAMA Network Open.*

eFigure 1. The PROTECT Intervention for the Prevention of Gaming Disorder and Unspecified Internet Use Disorder

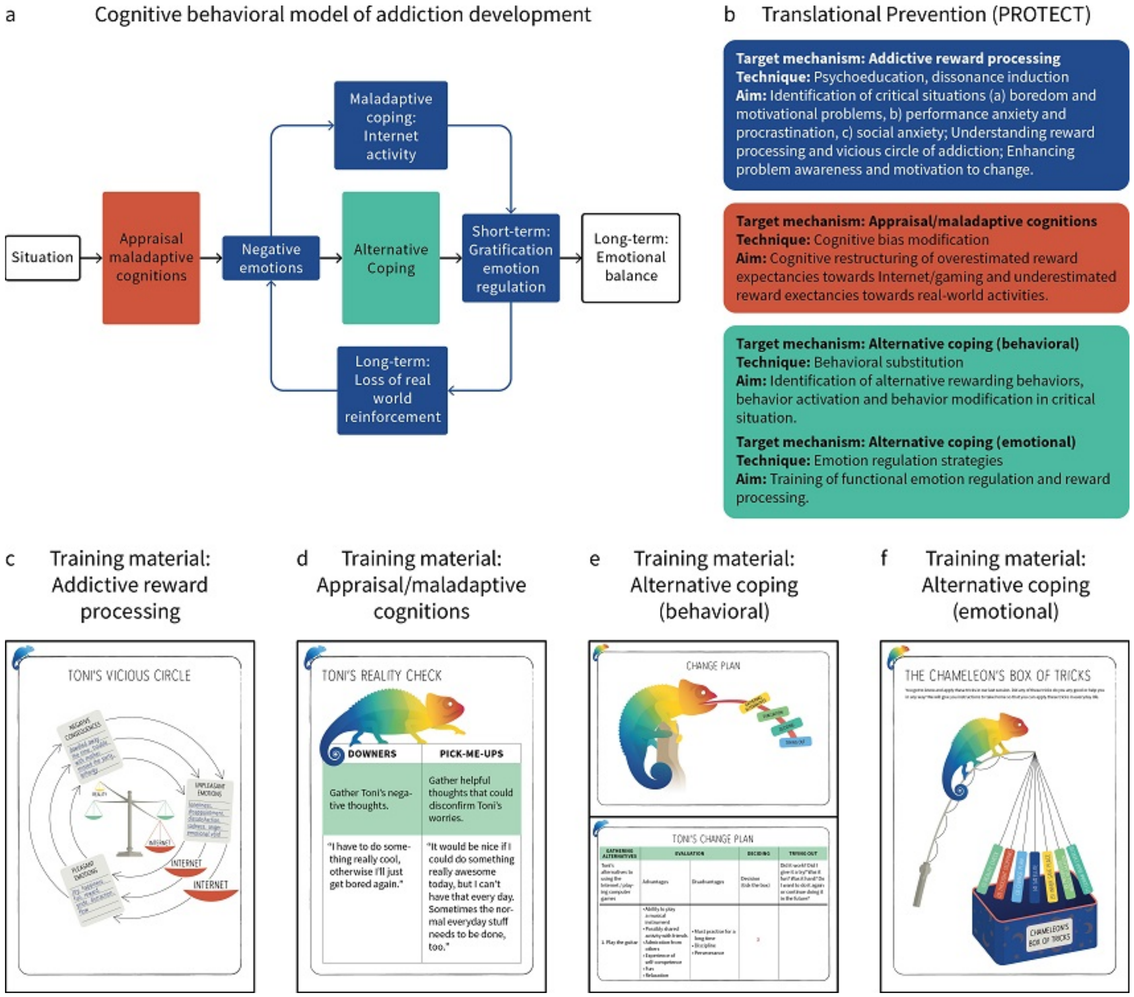

Note. 1a) The cognitive behavioral etiology model of gaming disorder and unspecified Internet use disorder (Lindenberg et al., 2020)[13]. 1b) Translation of target mechanisms and intervention techniques. 1c)-1f) Training material.

© 2022 Lindenberg K et al. *JAMA Network Open.*

eFigure 2. Flow of Participants for Incidence Analysis

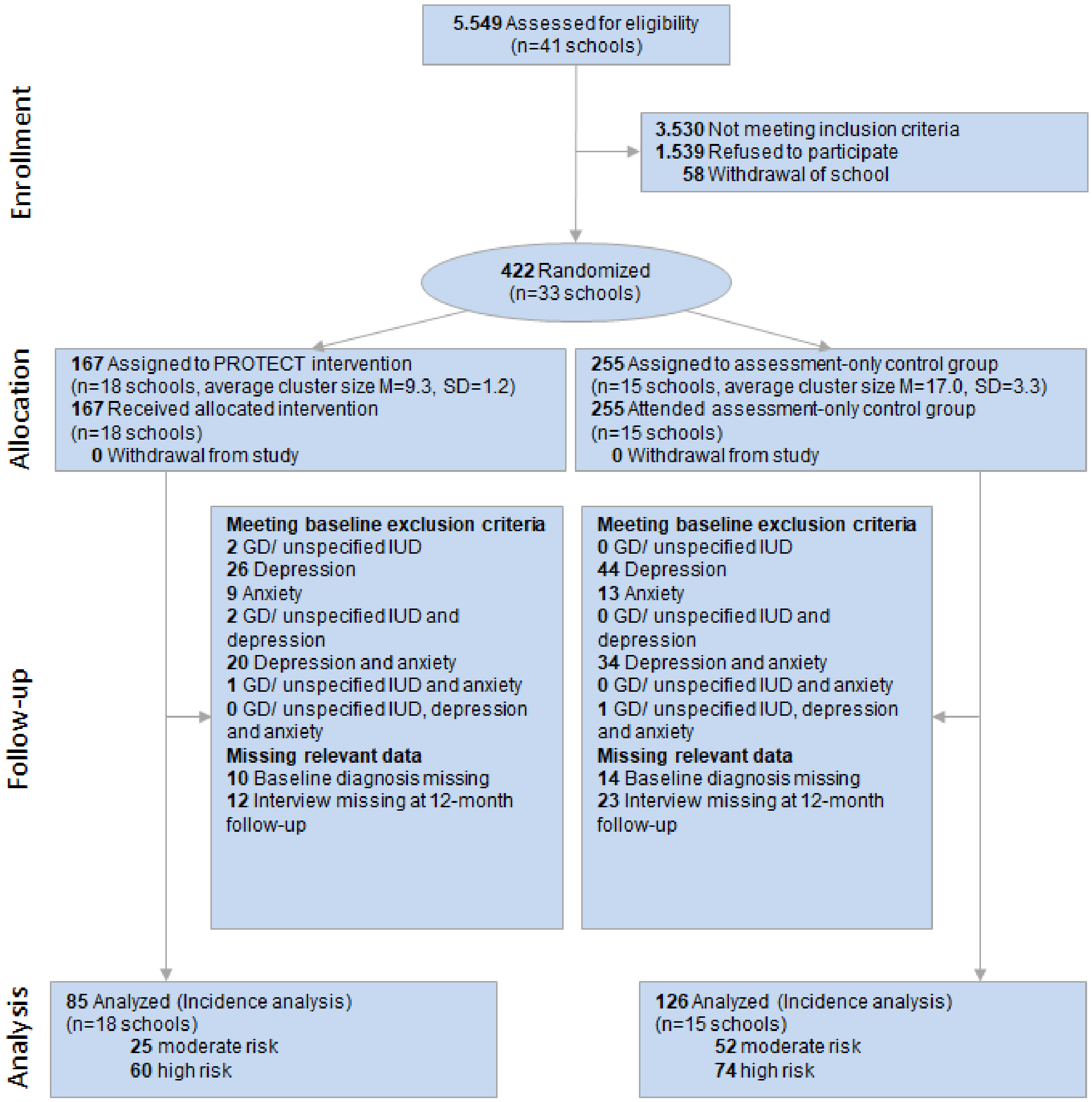

Note. GD=gaming disorder, unspecified IUD=unspecified Internet use disorder. *Moderate risk* is defined as 20≥CIUS≤23, *high risk* is defined as CIUS≥24. CIUS=compulsive Internet use scale.

© 2022 Lindenberg K et al. *JAMA Network Open.*

eFigure 3. Procrastination Symptom Changes Over 12 Months

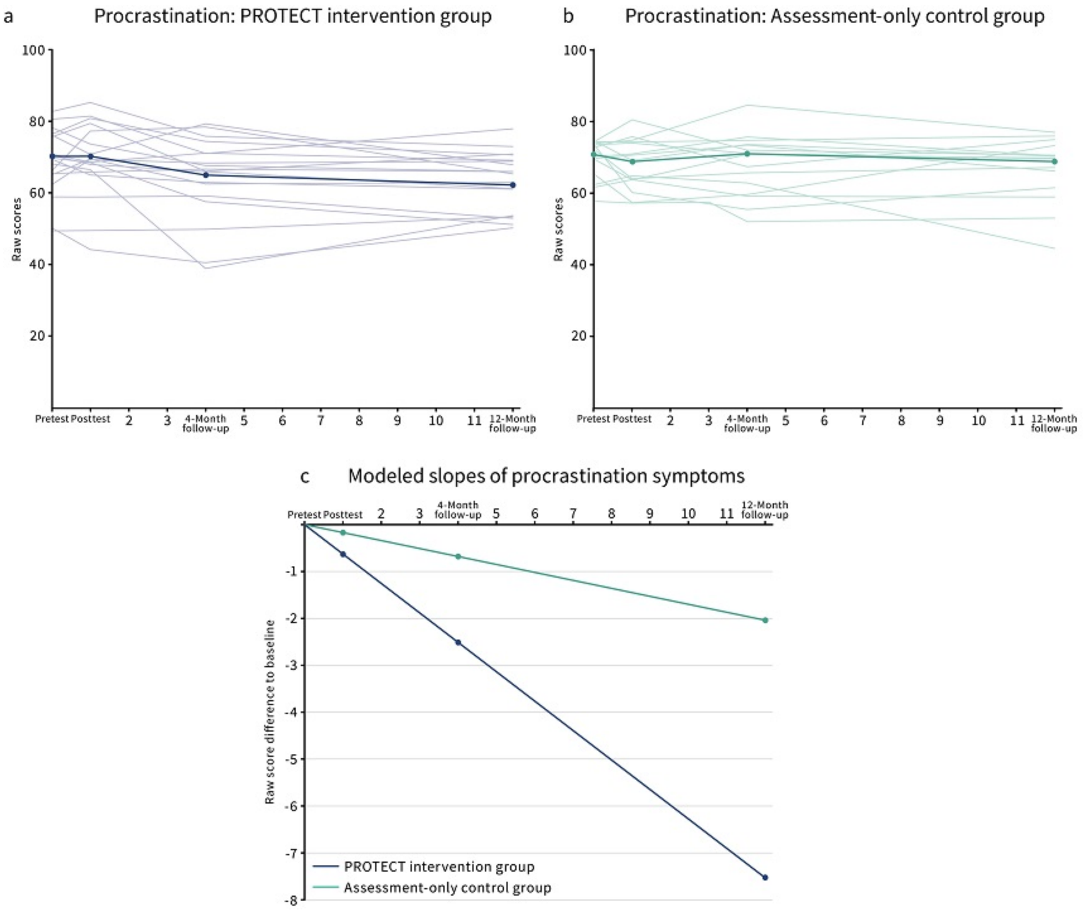

Note. 3a) Symptom courses in PROTECT intervention group (each line represents average score of one school) 3b) symptom in courses assessment-only control group, 3c) modeled symptom courses.

© 2022 Lindenberg K et al. *JAMA Network Open.*

eTable 1. Descriptive Statistics Separated by Group

	PROTECT group			Assessment-only control group			Group differences	
	n	%		*n*	%		*Chi*^{2}	*P*
Sex								
Male	79	47.3		114	44.7		.275 (*df*=1)	.600
Female	88	52.7		141	55.3			
School type								
low-level	32	19.2		20	7.8		11.966 (*df*=1)	.001
middle-level	41	24.6		19	7.5		24.193 (*df*=1)	<.001
high-level	51	30.5		99	38.8		3.023 (df=1)	.051
comprehensive school	10	6.0		8	3.1		2.008 (df=1)	.122
vocational track (low-level)	12	7.2		23	9.0		.446 (df=1)	.316
vocational track (high-level)	21	12.6		86	33.7		23.852 (df=1)	<.001
	M	*SD*		*M*	*SD*		*t*	*p*
Age	14.60	1.96		15.44	1.98		4.266 (*df*=420)	<.001
IA Screening (CIUS)	29.05	6.98		26.21	5.61		4.403 (*df*=300.946)	<.001
GD/ unspecified IUD (CSAS)	15.01	7.43		12.92	7.09		2.818 (*df*=396)	.005
Time spent online (hrs/day)	4.75	2.20		4.54	2.22		-.827 (*df*=336)	.409
Procrastination (APROF)	69.86	19.57		70.62	20.70		.362 (*df*=393)	.717
General Psychopathology (SDQ)	12.28	4.95		12.50	4.72		.454 (*df*=396)	.650
Depressive Symptoms (DIKJ)	14.69	6.94		15.01	7.09		.440 (*df*=395)	.660
Social Anxiety (SIAS)	24.52	12.00		26.24	13.38		1.306 (*df*=396)	.192
Performance Anxiety and School Anxiety (PHOKI)	6.83	3.91		7.04	3.60		.553 (*df*=394)	.581
Adaptive Emotion Regulation Strategies (FEEL-KJ)	6.37	1.28		6.45	1.25		.662 (*df*=394)	.508
Maladaptive Emotion Regulation Strategies (FEEL-KJ)	5.30	1.28		5.54	1.38		1.712 (*df*=394)	.088
Social and Learning Behavior (SSL)	86.05	17.76		91.31	14.52		3.088 (*df*=284.763)	.002
Self-Efficacy (SWE)	26.95	5.07		27.13	4.98		.354 (*df*=395)	.724

Note. The German school system comprises six secondary-school types, i.e., low-level schools, middle-level schools, high-level schools, comprehensive schools, vocational schools at low-level and vocational schools at high-level. GD=gaming disorder, unspecified IUD=unspecified Internet use disorder. CIUS=compulsive Internet use scale; CSAS=modified German video game dependency scale to assess GD/ unspecified IUD. APROF=procrastination scale; SDQ=general psychopathology scale; DIKJ=depression scale; SIAS=social anxiety scale; PHOKI=performance anxiety and school anxiety scale; FEEL-KJ adaptive=adaptive emotion regulation strategy scale; FEEL-KJ maladaptive=maladaptive emotion regulation strategy scale; SSL=social and learning behavior scale; SWE=self-efficacy scale.

© 2022 Lindenberg K et al. *JAMA Network Open.*

eTable 2. Descriptive Statistics and Effect Sizes of Primary Outcomes

Outcome Measure	Baseline	1-month FU	4-month FU	12-month FU	Baseline vs. 12-months
	M (SD)	*M (SD)*	*M (SD)*	*M (SD)*	*d*
**GD/ unspecified IUD Symptom Severity (CSAS)**					
PROTECT intervention group	13.74 (6.82)	14.46 (8.24)	12.09 (8.28)	9.20 (8.26)	0.67
Assessment-only control group	13.74 (6.81)	12.48 (7.15)	12.74 (7.91)	10.07 (6.89)	0.54

Note. Level-3 baseline differences were controlled. Level 3 baseline data (means) by school can be found in eTable3. GD=gaming disorder, unspecified IUD = unspecified Internet use disorder. CSAS= modified German video game dependency scale to assess GD/ unspecified IUD. *d* = Cohen's *d* statistic.

© 2022 Lindenberg K et al. *JAMA Network Open.*

eTable 3. Level 3 Baseline Data (Means) by School

School	CSAS *M*	APROF *M*	SDQ M	DIKJ *M*	SIAS *M*	PHOKI *M*	FEEL adapt. *M*	FEEL malad. *M*	SSL *M*	SWE *M*
1	10.83	69.50	15.38	15.00	28.44	6.83	6.11	5.28	79.50	25.83
2	12.50	67.77	15.17	17.33	28.67	7.85	6.60	5.23	83.33	25.00
3	22.50	62.00	9.50	8.00	13.00	4.00	6.26	5.15	105.50	32.50
4	15.67	62.17	12.00	14.00	29.67	8.00	6.14	4.52	94.50	27.17
5	11.50	73.50	14.88	17.50	19.97	6.00	6.20	6.02	82.75	28.00
6	14.00	70.50	11.25	11.75	26.00	3.00	6.27	5.03	79.75	25.25
7	14.86	73.32	12.40	13.50	26.13	7.26	6.84	4.98	90.29	28.32
8	16.17	64.73	12.07	17.42	25.73	8.67	5.82	5.51	83.06	26.60
9	19.66	70.19	14.41	16.38	23.92	6.67	6.09	5.08	72.77	24.25
10	11.12	74.54	12.63	16.03	25.19	6.80	6.68	5.96	89.46	28.12
11	19.24	72.55	13.40	18.00	30.80	7.40	6.50	6.16	80.60	24.60
12	12.70	61.45	12.00	14.39	27.87	7.20	6.34	5.14	88.45	27.75
13	18.83	76.00	12.00	10.83	20.17	6.50	6.68	5.15	93.50	28.00
14	13.24	70.05	10.54	12.48	28.67	7.03	6.30	5.40	95.62	27.67
15	11.63	69.38	11.11	11.58	19.56	5.94	6.72	5.16	95.31	29.69
16	14.99	69.07	10.44	13.16	25.00	6.70	6.73	5.36	85.80	28.29
17	11.30	58.60	11.15	12.60	20.18	4.40	6.25	4.57	96.80	29.20
18	13.21	73.74	12.95	16.33	24.67	7.11	6.46	5.79	95.48	25.68
19	12.20	65.80	10.40	12.80	21.40	5.00	6.76	5.18	90.40	27.80
20	13.71	82.29	14.79	17.79	26.14	7.14	6.22	6.16	89.93	25.53
21	11.57	57.57	14.14	14.14	27.86	7.86	5.89	5.75	87.71	27.29
22	14.13	75.75	11.75	14.75	30.13	7.75	5.83	5.40	88.38	27.25
23	12.15	69.39	11.25	14.59	25.54	6.82	6.25	5.59	96.33	25.80
24	17.60	65.04	12.05	17.82	24.96	8.55	5.83	5.19	78.27	24.57
25	16.71	77.86	16.71	16.71	33.29	8.71	7.26	5.39	70.86	24.29
26	13.17	49.17	15.17	11.79	8.00	5.33	5.87	4.37	87.53	27.33
27	16.57	80.17	11.36	15.71	33.07	6.00	6.82	5.81	86.43	28.43
28	17.19	72.14	16.71	21.57	22.00	6.57	5.86	6.19	85.00	29.57
29	10.65	74.22	12.29	13.78	24.98	6.83	6.82	5.83	95.94	26.83
30	13.40	65.20	14.00	16.00	25.18	7.50	5.93	4.56	86.06	25.61
31	17.00	74.14	13.14	17.86	32.43	7.29	6.38	6.11	88.57	26.14
32	15.25	75.25	9.25	15.50	30.00	10.00	6.06	5.70	89.08	25.75
33	6.00	50.00	9.00	5.50	15.33	4.50	7.01	4.43	107.00	31.00

Note. CSAS= modified German video game dependency scale to assess gaming disorder and unspecified Internet use disorder. APROF=procrastination scale; SDQ=general psychopathology scale; DIKJ=depression scale; SIAS=social anxiety scale; PHOKI=performance anxiety and school anxiety scale; FEEL adapt.=adaptive emotion regulation strategy scale; FEEL malad.=maladaptive emotion regulation strategy scale; SSL=social and learning behavior scale; SWE=self-efficacy scale.

© 2022 Lindenberg K et al. *JAMA Network Open.*

eTable 4. Parameter Estimates for Multilevel Linear Growth Model Examining GD/Unspecified IUD Symptom Reduction

			GD/ unspecified IUD Symptoms (CSAS-Score)				
Parameter			Model 0		Model 1		Model 2
Fixed Effects							
Initial Status	Intercept (γ_{00})		**12.49***** (0.45)		**13.91***** (0.46)		**12.76***** (0.56)
	PROTECT (γ_{01})						**2.47**** (0.84)
Rate of Change	Slope (γ_{10})				**-0.35***** (0.03)		**-0.30***** (0.04)
	PROTECT (γ_{11})						**-0.13*** (0.06)
Variance Components							
Level-1 residual variance	Within-Person (σ_{ε}^{2})		**30.65***** (1.34)		**26.97***** (1.18)		**26.86***** (1.18)
Level-2 residual variance	Initial Status (σ_{0}^{2})		**29.22***** (2.77)		**30.54***** (2.78)		**30.66***** (2.79)
Level-3 residual variance	Initial Status (σ_{2}^{2})		2.78 (1.54)		2.76 (1.51)		1.61 (1.31)
Model Fit Parameters							
-2 Log-Likelihood			9726.92		9596.64		9586.95
AIC			9734.92		9606.64		9600.95

Note. PROTECT=dummy coded group variable (1=PROTECT intervention group, 0=assessment-only control group). GD=gaming disorder, unspecified IUD=unspecified Internet use disorder, CSAS=modified German video game dependency scale to assess GD/ unspecified IUD. Standard errors are displayed in brackets. The rate of change displays the amount of change per month. **p* < .05, ***p* < .01, ****p* < .001.

© 2022 Lindenberg K et al. *JAMA Network Open.*

eTable 5. 12-Months Incidence Rates by Group, Stratified by Baseline Risk of Illness-Onset

		PROTECT intervention group	Assessment-only control group	*p*
Full-syndrome cases (GD) at 12-month follow-up				
Moderate risk at baseline	total	*n*=25	*n*=52	
	no	*n*=25 (100.0%)	*n*=52 (100.0%)	
	case	*n*=0 (00%)	*n*=0 (0.0%)	
High risk at baseline	total	*n*=60	*n*=74	
	no	*n*=60 (100.0%)	*n*=74 (100.0%)	
	case	*n*=0 (0.0%)	*n*=0 (0.0%)	
Full-syndrome cases (unspecified IUD) at 12-month follow-up				
Moderate risk at baseline	total	*n*=25	*n*=52	.245
	no	*n*=23 (92.0%)	*n*=51 (98.1%)	
	case	*n*=2 (8.0%)	*n*=1 (1.8%)	
High risk at baseline	total	*n*=60	*n*=74	.631
	no	*n*=56 (93.3%)	*n*=69 (93.2%)	
	case	*n*=4 (6.7%)	*n*=5 (6.8%)	
Subthreshold cases (GD) at 12-month follow-up				
Moderate risk at baseline	total	*n*=25	*n*=52	.526
	no	*n*=23 (92.0%)	*n*=49 (94.0%)	
	case	*n*=2 (8.0%)	*n*=3 (5.8%)	
High risk at baseline	total	*n*=60	*n*=74	
	no	*n*=59 (98.3%)	*n*=70 (94.6%)	.255
	case	*n*=1 (1.7%)	*n*=4 (5.4%)	
Subthreshold cases (unspecified IUD) at 12-month follow-up				
Moderate risk at baseline	total	*n*=25	*n*=52	.406
	no	*n*=22 (88.0%)	*n*=43 (82.7%)	
	case	*n*=3 (12.0%)	*n*=9 (17.3%)	
High risk at baseline	total	*n*=60	*n*=74	
	no	*n*=53 (88.3%)	*n*=60 (81.1%)	.182
	case	*n*=7 (11.7%)	*n*=14 (18.9%)	
Any subthreshold or full-syndrome cases (GD/unspecified IUD) at 12-month follow-up				
Moderate risk at baseline	total	*n*=25	*n*=52	.420
	no	*n*=18 (72.0%)	*n*=40 (76.9%)	
	case	*n*=7 (28.0%)	*n*=12 (23.1%)	
High risk at baseline	total	*n*=60	*n*=74	
	no	*n*=49 (81.7%)	*n*=52 (70.3%)	.093
	case	*n*=11 (18.3%)	*n*=22 (29.7%)	

Note. GD=gaming disorder, unspecified IUD = unspecified Internet use disorder. *Moderate risk* is defined as 20≥CIUS≤23, *high risk* is defined as CIUS≥24. CIUS=compulsive Internet use scale; cases and healthy individuals are presented in total numbers; incidence rates are displayed in brackets. Full-syndrome cases=5 or more GD or unspecified IUD criteria. Subthreshold cases=3 or 4 GD or unspecified IUD criteria. Any subthreshold or full-syndrome cases = anyone meeting 3 or more GD and/ or unspecified IUD criteria.

© 2022 Lindenberg K et al. *JAMA Network Open.*

eTable 6. Correlation Matrix of GD/Unspecified IUD Symptoms With Comorbid Symptoms at Baseline

	1	2	3	4	5	6	7	8	9	10
GD/ unspecified IUD (CSAS)	-									
Procrastination (APROF)	.266**	-								
General Psychopathology (SDQ)	.326**	.379**	-							
Depressive Symptoms (DIKJ)	.269**	.485**	.690**	-						
Social Anxiety (SIAS)	.275**	.349**	.453**	.522**	-					
Performance and School Anxiety (PHOKI)	.206**	.260**	.328**	.426**	.494**	-				
Adaptive Emotion Regulation (FEEL-KJ)	-.132**	-.111*	-.270**	-.374**	-.191**	-.115*	-			
Maladaptive Emotion Regulation (FEEL-KJ)	.244**	.340**	.494**	.569**	.410**	.246	-.193**	-		
Social and Learning Behavior (SSL)	-.311**	-.345**	-.410**	-.458**	-.290**	-.191**	.263**	-.108*	-	
Self-Efficacy (SWE)	-.165**	-.240**	-.394**	-.576**	-.470**	-.393**	.404**	-.375**	.400**	-

Note. GD=gaming disorder, unspecified IUD= unspecified Internet use disorder. CSAS= modified German video game dependency scale to assess GD/ unspecified IUD. APROF=procrastination scale; SDQ=general psychopathology scale; DIKJ=depression scale; SIAS=social anxiety scale; PHOKI=performance anxiety and school anxiety scale; FEEL-KJ adaptive=adaptive emotion regulation strategy scale; FEEL-KJ maladaptive=maladaptive emotion regulation strategy scale; SSL=social and learning behavior scale; SWE=self-efficacy scale. **p* < .05, ***p* < .01.

© 2022 Lindenberg K et al. *JAMA Network Open.*

eTable 7. Parameter Estimates for Multilevel Linear Growth Model Examining Procrastination Symptom Reduction

		Procrastination (APROF-score)		
Parameter		Model 0	Model 1	Model 2
Fixed Effects				
Initial Status	Intercept (γ_{00})	**68.13*****<br>(1.17)	**69.62*****<br>(1.19)	**70.09*****<br>(1.51)
	PROTECT (γ_{01})			-.58<br>(2.27)
Rate of Change	Slope (γ_{10})		**-.35*****<br>(0.69)	-.17<br>(0.09)
	PROTECT (γ_{11})			**-.46*****<br>(0.14)
Variance Components				
Level-1 residual variance	Within-Person (σ_{ε}^2)	**153.40*****<br>(6.77)	**149.62*****<br>(6.60)	**148.23*****<br>(6.54)
Level-2 residual variance	Initial Status (σ_0^2)	**278.40*****<br>(23.46)	**280.41*****<br>(23.51)	**281.79*****<br>(23.67)
Level-3 residual variance	Initial Status (σ_2^2)	14.75<br>(10.76)	13.58<br>(10.38)	6.61<br>(9.76)
Model Fit Parameters				
-2 Log-Likelihood		12232.35	12206.91	12195.36
AIC		12240.38	12216.91	12209.36

Note. APROF=procrastination scale. PROTECT=dummy coded group variable (1=PROTECT intervention group, 0=assessment-only control group). Standard errors are displayed in brackets. The rate of change displays the amount of change per month. *$p < .05$, **$p < .01$, ***$p < .001$.

eTable 8. Descriptive Statistics and Effect Sizes of Secondary Outcomes

Outcome Measure	Baseline	1-month FU	4-month FU	12-month FU	Baseline vs. 12-month FU
	M (SD)	*M (SD)*	*M (SD)*	*M (SD)*	*d*
Procrastination (APROF)					
PROTECT	70.32 (18.15)	70.16 (18.92)	65.68 (19.33)	63.84 (20.79)	0.357
Controls	70.32 (20.18)	68.53 (21.19)	70.39 (21.88)	68.63 (20.19)	0.084
General Psychopathology (SDQ)					
PROTECT	12.42 (4.55)	12.92 (5.91)	11.51 (5.32)	10.73 (5.00)	0.372
Controls	12.42 (4.55)	11.84 (4.81)	11.52 (4.92)	11.29 (5.05)	0.247
Depressive Symptoms (DIKJ)					
PROTECT	14.89 (6.38)	14.08 (7.33)	12.87 (7.22)	12.48 (7.59)	0.378
Controls	14.89 (6.86)	13.68 (7.34)	13.51 (7.58)	12.94 (7.38)	0.284
Social Anxiety (SIAS)					
PROTECT	25.56 (10.97)	24.83 (12.07)	23.36 (11.76)	22.73 (12.29)	0.258
Controls	25.56 (13.20)	23.57 (12.47)	23.71 (13.23)	22.68 (12.82)	0.219
Performance Anxiety and School Anxiety (PHOKI)					
PROTECT	6.96 (3.61)	6.64 (3.65)	6.43 (3.38)	5.80 (3.33)	0.320
Controls	6.96 (3.59)	6.28 (3.72)	6.21 (3.69)	5.97 (3.79)	0.276
Adaptive emotion regulation strategies (FEEL-KJ adaptive)					
PROTECT	6.42 (1.21)	6.50 (1.47)	6.36 (1.33)	6.33 (1.45)	0.073
Controls	6.42 (1.21)	6.44 (1.32)	6.49 (1.22)	6.28 (1.32)	0.113
Maladaptive emotion regulation strategies (FEEL-KJ maladaptive)					
PROTECT	5.44 (1.21)	5.40 (1.17)	5.32 (1.13)	5.21(1.16)	0.192
Controls	5.44 (1.30)	5.40 (1.23)	5.47 (1.30)	5.31 (1.25)	0.100
Social and Learning Behavior (SSL)					
PROTECT	89.23 (15.78)	91.96 (15.47)	91.45 (18.91)	92.18 (16.65)	-0.187
Controls	89.23 (13.80)	90.57 (14.79)	90.40 (15.20)	92.77 (14.63)	-0.257
Self-Efficacy (SWE)					
PROTECT	27.06 (4.65)	27.62 (5.11)	28.18 (4.87)	27.93 (4.85)	-0.186
Controls	27.06 (4.85)	27.31 (5.56)	27.95 (5.39)	27.68 (5.28)	-0.128

Note. Level-3 baseline differences were controlled. Level-3 baseline data (baseline means by school) can be found in Supplementary Table ST2. APROF=procrastination scale; SDQ=general psychopathology scale; DIKJ=depression scale; SIAS=social anxiety scale; PHOKI=performance anxiety and school anxiety scale; FEEL-KJ adaptive=adaptive emotion regulation strategy scale; FEEL-KJ maladaptive=maladaptive emotion regulation

© 2022 Lindenberg K et al. *JAMA Network Open.*

strategy scale; SSL=social and learning behavior scale; SWE=self-efficacy scale. *d* = Cohen's *d* statistic.

© 2022 Lindenberg K et al. *JAMA Network Open.*

eTable 9. Results of Fixed Effects Parameters for Secondary Outcome Measures

Variable	Parameter	Estimate	*SE*	*t* value	*p* value
Procrastination	Intercept (γ_{00})	70.085	1.514	46.288	<0.001
(APROF)	Time (γ_{10})	-0.170	0.896	-1.895	0.058
	PROTECT*Time (γ_{11})	-0.458	0.141	-3.236	0.001
General Psychopathology	Intercept (γ_{00})	12.263	0.337	36.370	<0.001
(SDQ)	Time (γ_{10})	-0.867	0.021	-4.077	<0.001
	PROTECT*Time (γ_{11})	-0.049	0.033	-1.466	0.143
Depressive Symptoms	Intercept (γ_{00})	14.507	0.598	24.248	<0.001
(DIKJ)	Time (γ_{10})	-0.136	0.027	-5.113	<0.001
	PROTECT*Time (γ_{11})	-0.627	0.042	-1.484	0.138
Social Anxiety	Intercept (γ_{00})	25.139	0.741	33.918	<0.001
(SIAS)	Time (γ_{10})	-0.168	0.051	-3.308	0.001
	PROTECT*Time (γ_{11})	-0.155	0.080	-1.932	0.054
Performance Anxiety and School Anxiety	Intercept (γ_{00})	6.722	0.213	31.623	<0.001
(PHOKI)	Time (γ_{10})	-0.064	0.016	-3.860	<0.001
	PROTECT*Time (γ_{11})	-0.043	0.026	-1.654	0.098
Adaptive Emotion Regulation Strategies	Intercept (γ_{00})	6.471	0.086	75.047	<0.001
(FEEL-KJ)	Time (γ_{10})	-0.009	0.006	-1.352	0.177
	PROTECT*Time (γ_{11})	-0.007	0.010	-0.732	0.464
Maladaptive Emotion Regulation Strategies	Intercept (γ_{00})	5.506	0.093	59.206	<0.001
(FEEL-KJ)	Time (γ_{10})	-0.014	0.006	-2.454	0.014
	PROTECT*Time (γ_{11})	-0.005	0.009	-0.502	0.616
Social and Learning Behavior	Intercept (γ_{00})	90.337	1.759	51.352	<0.001
(SSL)	Time (γ_{10})	0.290	0.069	4.225	<0.001
	PROTECT*Time (γ_{11})	-0.128	0.109	-1.179	0.239
Self-Efficacy	Intercept (γ_{00})	27.399	0.428	63.949	<0.001
(SWE)	Time (γ_{10})	0.048	0.025	1.907	0.057
	PROTECT*Time (γ_{11})	0.289	0.040	0.721	0.471

Note. PROTECT=dummy coded group variable (1=PROTECT intervention group, 0=assessment-only control group). The time parameter is scaled in months.

© 2022 Lindenberg K et al. *JAMA Network Open.*

eReferences

1. Lindenberg K, Halasy K, Schoenmaekers S. A randomized efficacy trial of a cognitive-behavioral group intervention to prevent Internet Use Disorder onset in adolescents: The PROTECT study protocol. *Contemporary Clinical Trials Communications*. 2017;6:64-71. doi:10.1016/j.conctc.2017.02.011

2. Höcker A, Engberding M, Rist F. *Prokrastination: Ein Manual Zur Behandlung Des Pathologischen Aufschiebens*. Hogrefe Verlag; 2013.

3. Goodman R. The Strengths and Difficulties Questionnaire (SDQ). In: VandeCreek L, Jackson TL, VandeCreek L(Jackson TL(eds. *Innovations in clinical practice: Focus on children & adolescents*. Professional Resource Press/Professional Resource Exchange; 2003:109-111.

4. Stiensmeier-Pelster J, Schürmann M, Duda K. *Depressions-Inventar Für Kinder Und Jugendliche:(DIKJ)*. Hogrefe; 2000.

5. Kovacs M. The Children's Depression, Inventory (CDI). *Psychopharmacol Bull*. 1985;21(4):995-998.

6. Mattick RP, Clarke J. Development and validation of measures of social phobia scrutiny fear and social interaction anxiety1. *Behaviour Research and Therapy*. 1998;36(4):455-470. doi:10.1016/S0005-7967(97)10031-6

7. Stangier -U, Heidenreich -T, Berardi A, Golbs -U, Hoyer -J. *Social Interaction Anxiety Scale - deutsche Fassung*. 1999.

8. Döpfner M, Schnabel M, Goletz H, Ollendick T. *Phobiefragebogen Für Kinder Und Jugendliche: PHOKI*. Hogrefe; 2006.

9. Ollendick TH. *Reliability and Validity of the Revised Fear Survey Schedule for Children (FSSC-R)*. 1983.

10. Grob A, Smolenski C. *FEEL-KJ: Fragebogen Zur Erhebung Der Emotionsregulation Bei Kindern Und Jugendlichen*; 2005.

11. Petermann U, Petermann F. *Schülereinschätzliste Für Sozial-Und Lernverhalten: SSL*. Hogrefe; 2014.

12. Schwarzer R, Jerusalem M. *Skalen Zur Erfassung Von Lehrer- Und Schülermerkmalen: Dokumentation Der Psychometrischen Verfahren Im Rahmen Der Wissenschaftlichen Begleitung Des Modellversuchs Selbstwirksame Schulen*; 1999.

13. Lindenberg K, Kindt S, Szàsz-Janocha C. *Internet Addiction in Adolescents: The PROTECT Program for Evidence-Based Prevention and Treatment*. Springer; 2020.

© 2022 Lindenberg K et al. *JAMA Network Open.*
