# Supplementary material for: Effectiveness of Cognitive Behavioral Therapy–Based Intervention in Preventing Gaming Disorder and Unspecified Internet Use Disorder in Adolescents: A Cluster Randomized Clinical Trial
Source: JAMA Netw Open. 2022 Feb 18;5(2):e2148995. doi: 10.1001/jamanetworkopen.2021.48995 (PMC8857686; doi:10.1001/jamanetworkopen.2021.48995)
Supplement: Supplement 2. — Trial Protocol [file jamanetwopen-e2148995-s002.pdf]

Study Protocol

Efficacy of a school-based Cognitive-Behavioral Group Intervention program to prevent Internet addiction.

Acronym: PROTECT

Principle Investigator:
Jun.-Prof. Dr. Katajun Lindenberg
Pädagogische Hochschule Heidelberg
Institut für Psychologie
Juniorprofessur für Entwicklungspsychologie
Keplerstr. 87
D-69120 Heidelberg

Heidelberg, 07.07.2015

1 General information

1.1 Applicant (Principle Investigator)

Jun.-Prof. Dr. Katajun Lindenberg, Junior Professor for Developmental Psychology, Faculty of Education and Social Sciences, Institute of Psychology, University of Education Heidelberg

1.2 Topic

Investigation of the efficacy of a school-based program for the prevention of Internet addiction and video game dependency

1.3 Acronym

PROTECT (Acronym for "Professioneller Umgang mit technischen Medien")

1.4 Field of expertise and field of work

Field: developmental and educational psychology

Field of work: quantitative-empirical psychological prevention research in schools

1.5 Application period

Start of funding: 01.05.2015

expected duration: 3 years

1.6 Summary

Background. The reduction of prevalence rates of Internet Use Disorder (IUD) and its effective treatment are at high priority in both public health and educational policies. School-based preventive interventions facilitate a low-threshold approach for individuals

with IUD, who are typically characterized by high therapy avoidance. Moreover, indicated approaches which target adolescents at high-risk show larger effects than universal prevention programs. Simultaneously, they reduce unnecessary burden for the majority of high-school students that is not at-risk. The PROTECT group intervention for indicated prevention of IUD in school settings was developed based on these preventive strategies.

Methods. Three-hundred and forty adolescents, aged 12-18 years, from 40 secondary schools in Germany, screened for high-risk of IUD onset, are randomly assigned to a) PROTECT preventive intervention group or b) assessment only control group. The tested intervention consists of a cognitive-behavioral 4-session brief-protocol. Follow-up assessments are at 1, 4 and 12 months after admission. Primary outcome is the 12-months incidence rate of IUD. Secondary outcomes are the reduction of IUD and comorbid symptoms as well as the promotion of problem solving, cognitive restructuring and emotion regulation skills.

Conclusion. The indicated preventive intervention PROTECT follows the APA-guidelines for psychological prevention, i.e., it is theory- and evidence-based and addresses both risk-reduction and strength-promotion, it considers current research and epidemiology and ethical standards such as professional secrecy and is designed as a systemic intervention at the school-level. It is expected that the intervention decreases risk of IUD onset (incidence rate).

Trial registration. ClinicalTrials.gov: NCT02907658

2. State of research

The prevention of illness onset has highest priority in mental disorders [1]. Increasing numbers of patients with Internet Use Disorder (IUD) underline the emerging public health challenge. Although IUD prevalence rates are highly inconsistent and range from 0.8% to 26.7% depending on the definition of IUD and its measurement [2], the need to develop effective preventive interventions is beyond dispute. “Internet Gaming Disorder” (IGD), which has recently been included in the DSM-5 [3], has been found to affect 1.2% of adolescents in representative community samples [4,5]. IGD diagnostic criteria explicitly exclude non-gaming subtypes due to lack of evidence. However, non-gaming IUD has been found to be associated with similar levels of impairment [6] [7]. The worldwide IUD prevalence estimate (gaming and non-gaming subtypes) based on a meta-analysis is about 6.0% [8]. Even subthreshold pathology, which afflicts 13.3% of adolescent boys and girls [9], produces severe psychosocial distress and increased risk for comorbid psychopathology [10].

Impairment caused by IUD comprises educational failure and reduced academic perspectives [11,12], health problems and functional imairment [13]. In addition, individuals with IUD are at high risk for mental comorbidities [14], such as depressive disorders, attention-deficit disorders and anxiety disorders [10,11,15]. Finally, societal costs for covering treatment and the consequences of academic failure are expected to be enormous.

Public health calls for effective IUD screening, preventive interventions and treatments emphasize the actual low evidence of IUD interventions in terms of efficacy (effect), cost-effectiveness (costs / effect) and public-health-impact (reach x effect / costs) [3,16].

Since target groups of preventive interventions typically show limited treatment motivation and specifically IUD is characterized by high therapy avoidance, it is crucial to offer low-threshold interventions, which can be implemented on a large-scale basis. Therefore, systemic approaches such as school-based prevention programs are of particular relevance [17]. Moreover, indicated interventions which target adolescents at high-risk show larger effects than universal prevention programs [18]. Simultaneously, they reduce unnecessary burden for the majority of high-school students that is not at-risk [19].

Evidence shows no systematic differences in efficacy between short and long-term behavioral preventive interventions. However, short interventions show enhanced dose-response ratios and thus superiority in school settings in terms of health economics and educational policy [18,20].

3. Methods and Design

3.1. Design and randomization

The study is designed as a prospective randomized controlled 2-armed intervention trial. Participants are randomly assigned to one of two conditions: an intervention group that receives the preventive intervention PROTECT and an assessment-only control group. Schools are considered as units of randomization (cluster randomization). Randomization is stratified by school type as defined by the German school system (Gymnasium, Realschule, Hauptschule) and conducted by an independent person. We expect a low intraclass correlation, thus, the cluster-randomization is not expected to interfere with the power calculation.

The study examines the effects of the preventive intervention on primary and secondary outcomes at baseline, 1-month follow-up, 4-months follow-up, and 12-months

follow-up (see Figure 1). Informed written consent is obtained from participants and their primary caregivers prior to enrolment. All data are cryptographically secured with codes consisting of random numbers and letters. Re-identification is only possible through the encryption key. Ethical approval was obtained from the University of Education Heidelberg Research Ethics Committee on September 3, 2015. Approval from the Regierungspräsidium Karlsruhe was obtained on October 19, 2015. The trial has been registered on April 18, 2016 at the University of Education Heidelberg research Database (PHHD-PROTECT-101030906). In addition, it has been registered on September 9, 2016 at clinicaltrials.gov (ClinicalTrials.gov Identifier: NCT02907658).

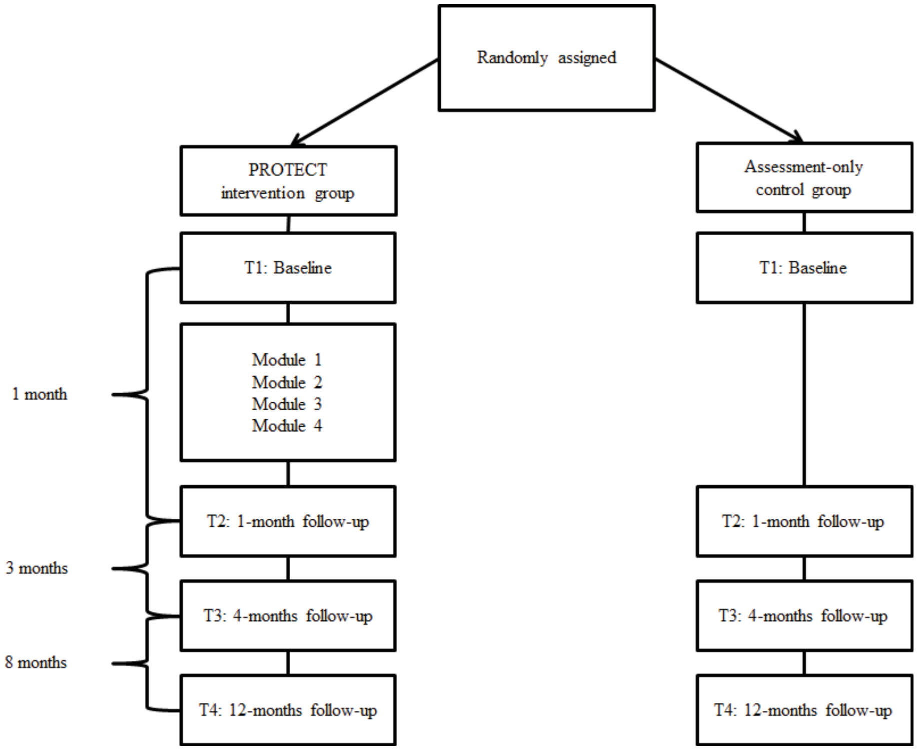

Figure 1. Design and Procedure.

3.2. Participants and procedure

Participants are recruited from high schools from September 2015 to December 2016 in the district “Metropolregion Rhein-Neckar”. Schools are approached directly by telephone, e-mail, information brochures and informative meetings. Moreover, key

persons such as school psychologists, school counselors and prevention teachers are involved in the recruitment process. School principals, teachers, parents and students receive detailed information about the relevance, aim and procedure of the study including information on randomization. The project is funded by the Dietmar Hopp Foundation from May 2015 to April 2018. Final results are expected in February 2018 (see Figure 2).

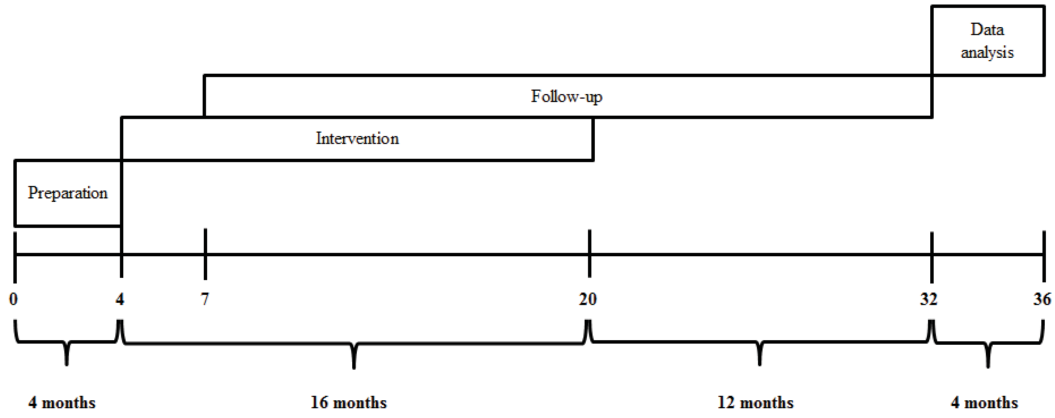

Figure 2. Time Schedule.

> The preparation period is used to train professionals delivering the intervention, recruit schools and obtain legal permissions. The intervention period includes recruitment, enrollment and intervention delivery. The follow-up period was chosen to allow 12-month follow-up assessments. Data analysis is planned after final data collection is completed.

Before enrolment, an anonymous screening procedure using the Compulsive Internet Use Scale [9,21] identifies students at-risk for IUD. Further inclusion and exclusion criteria are presented in Table 1. Because the main outcome is onset of threshold and subthreshold IUD, all participants are excluded who meet DSM-5 criteria for IGD [3] at baseline, extended to both gaming and non-gaming internet activities. Moreover, participants meeting criteria for comorbid depression or anxiety disorder are excluded. These adolescents are referred to specialized treatment.

Table 1. *Inclusion and exclusion criteria.*

**Inclusion criteria**	**Exclusion criteria**
Adolescents aged 12 to 18 years	Current IUD diagnosis or treatment
Written informed consent	Comorbid depression
High-risk for IUD (CIUS ≥ 20)	Comorbid anxiety disorder (social phobia or performance anxiety)

Study administrators coordinate all appointments for training and assessments in cooperation with the school management. The preventive intervention group participates in the PROTECT training in groups of 6-10 adolescents for 4 subsequent weekly sessions (à 90 minutes). The group intervention is delivered by a team of two experienced professionals who completed a PROTECT intervention delivery training. We ensure that at least one of the trainers delivering the intervention is a graduated psychologist, accompanied by an undergraduate student. The trainers are regularly supervised in team meetings. We use clinical diagnostic interviews to assess IGD according to the DSM-5 (gaming and non-gaming subtypes) at 12-months follow-up (primary outcome). In addition, assessments at baseline and follow-ups include psychometric data on IUD, potential risk factors, comorbid psychopathology, and school-related consequences such as grades and truancy (secondary outcomes). We expect a significant reduction of incidence rates of IUD in the high-risk intervention group compared to the assessment-only control group at 12 months after the preventive intervention.

3.3. Sample size and power calculation

Assuming an effect which reduces the incidence rate in the high-risk group (approximately 14% of total population) from 36% to 24% at 12-months follow-up, with an $\alpha = 0.05$ (two-sided), a total sample of high risk students of N = 340 (170 per group) is needed to assure a power of 80% ($ß = 0.2$). This corresponds to an empirical population of N = 2430 (N = 1215 per group) unselected high-school students. Accounting for a 25% drop-out rate, a total sample of N = 3240 is addressed for screening. In average, this corresponds to 170 classes in 43 schools (see Figure 3).

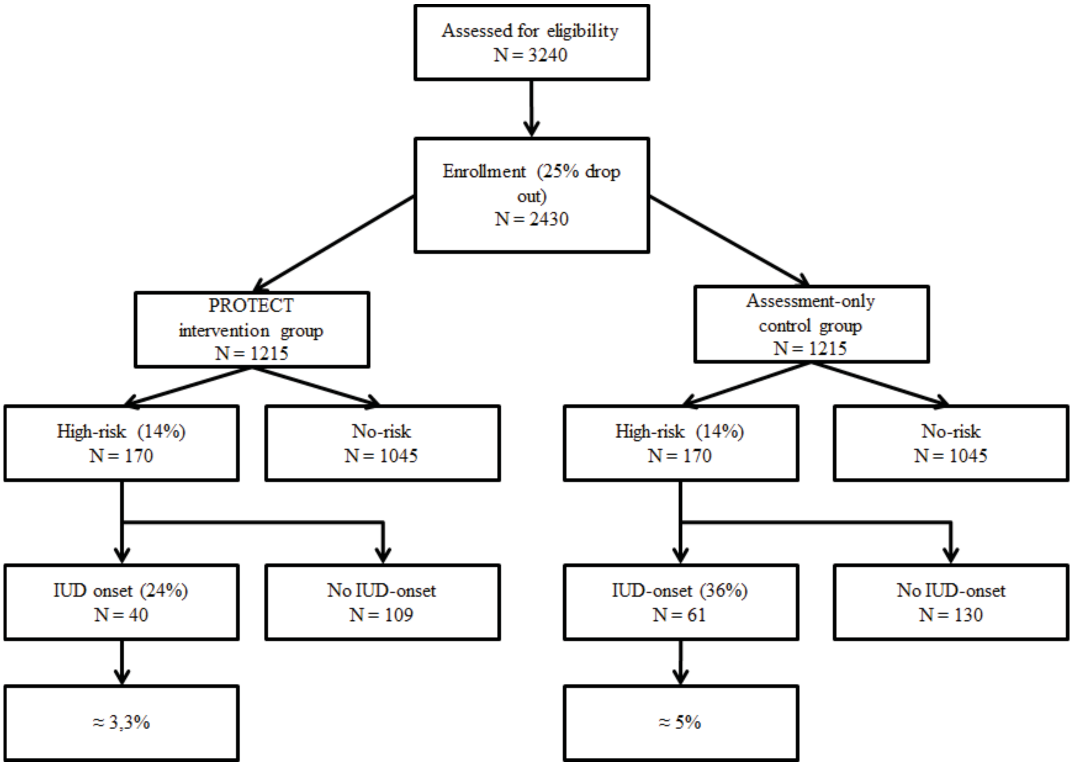

Figure 3. Planned sample size.

3.4. Intervention

3.4.1. The prevention program

The preventive short intervention PROTECT for group settings comprises 4 modules focusing (1) boredom and motivational problems, (2) procrastination and performance

anxiety, (3) social interaction and (4) emotion regulation. These 4 domains are derived from current empirical findings about risk factors of IUD [22,23] PROTECT is based on cognitive behavioral (CB) interventions [24,25] such as (1) psychoeducation, (2) cognitive restructuring (identification and modification of dysfunctional beliefs), (3) behavior modification (improving problem solving skills, training of functional behavior and contingency management) as well as (4) improving emotion regulation (training of sensory, imaginative and mindfulness based techniques).

Rational-emotive stories [26,27] are used to illustrate possible pathways to IUD. Subsequently, dysfunctional beliefs, behaviors and emotional dysregulations are addressed by the above mentioned CB-therapy techniques.

Cognitive Restructuring. PROTECT is based on a CB model, assuming that cognitive biases such as overgeneralization, catastrophizing or demandingness cause negative emotions [25,28,29] and that excessive Internet use is a conditioned, maladaptive strategy to cope with negative affect. The CB disease model is elaborated in each session, demonstrating how positive consequences (conditioning.g. distraction) of the internet activity result in maintenance of a vicious circle (operant conditioning) which leads to harmful effects (e.g. poor grades) in the long run. PROTECT does not aim at sanctioning the internet activity but at scrutinizing cognitive distortions on the basis of empirical ("How likely is it?"), logical ("Is there any proof?"), normative ("Am I allowed to do this?"), functional ("Is it in line with my goal?"), and hedonistic ("Does it increase my well-being?") criteria. Dysfunctional beliefs are then replaced by more realistic assumptions [28].

Behavior Modification. Behavior modification techniques in PROTECT include improvement of problem solving skills, training of functional behavior and contingency management. The training of problem solving skills [30,31] is a CB technique that

follows a structured sequence of actions to elaborate a solution for specific problems. This technique aims at adopting a structured set of actions, which can be transferred to the solution of other problems. A problem solving sequence is typically divided into (a) a phase of orientation, problem definition and the generation of alternative solutions ("generate solutions"), (b) an evaluation phase of each possible solution ("evaluate solutions"), (c) a decision for one option "(pick a solution") and (d) a phase of action ("plan implementation"), in which the chosen solution is realized and its effectiveness is evaluated [32]. Several studies verified the effectiveness of problem solving trainings for different problem areas for adults [33] and adolescents [30].

Emotion regulation. To improve emotion regulation skills, PROTECT bases on techniques for psychophysiological emotion regulation which have been proven effective in combination with CB therapy in prevention and therapy for a variety of indications such as anxiety disorders, depression, chronic pain and attention deficit disorder [34]. Specifically, PROTECT includes sensory methods, imaginative methods and mindfulness-based techniques.

3.5. Assessments

Questionnaires assessing behavioral and psychological outcomes are completed at school during class hours. The completion of all questionnaires takes approximately 45 minutes per assessment. Beyond psychometric data, clinical outcomes at 12-months follow-up are assessed in a clinical interview by a blinded rater.

3.5.1. Sociodemographic data and Internet Use

At baseline, sociodemographic data are collected. The self-report demographics questionnaire includes information on gender, age, type of school, grades in mathematics

and German, country of birth and absence rates from school. Internet related information regarding the average time spent online on weekdays and weekends and the main internet activities (surfing, gaming or chatting) are assessed. Participants are asked whether they have searched counselling or treatment for IUD in the past.

3.5.2. Screening for risk of Internet Use Disorder onset

In order to assess risk for IUD at screening, we use the German version of the Compulsive Internet Use Scale [9,21] . This instrument was constructed according to the diagnostic criteria for dependency and obsessive compulsive disorders [35]. The 14 items of the questionnaire are rated on a 5-point Likert scale from 0 to 4 (“never”, “rarely”, “sometimes”, “often”, “very often”) with a total score ranging from 0 to 56. It has been argued that a total score of 24 and higher is recommended to identify cases with a sensitivity of 70% [36]. Aiming at achieving a high sensitivity and simultaneously limiting the total number needed to treat we decided to use a more liberal cut-off score of 20 to select participants with elevated risk, which constitute about 30% of the total population. The CIUS shows a high internal consistency reliability coefficient (Cronbach’s $\alpha = .89$) as well as a high internal validity due to its similarity to the diagnostic criteria of IGD [3,37].

3.5.3. Primary Outcome

Incidence of IUD. Participants in both arms are diagnosed at 12-months follow-up using a clinical interview based on the criteria of IGD as proposed in DSM-5 section 3 [3]: (1) preoccupation, (2) withdrawal, (3) tolerance, (4) unsuccessful attempts to control, (5) loss of interest in other activities, (6) continued excessive use despite problems, (7) deception (8) maladaptive coping, (9) loss of relationship, job, or educational or career

opportunities. To assess both gaming and non-gaming subtypes, we extend the IGD definition to IUD. Because we excluded all participants meeting IUD-criteria at baseline, the interview allows for determination of the 12-months incidence rate. A reduction of incidence rates is regarded as the strongest empirical proof for the preventive effectiveness of a prevention program.

Prevalence of IUD. To assess changes in IUD prevalence over a 1-year period, we use the German Video Game Dependency Scale [38]. The questionnaire includes all 9 diagnostic criteria of IGD as defined in DSM-5 [3]. These criteria are assessed by 2 items each (18 in total), which are rated on a 4-point Likert scale from 0 to 3 ("strongly disagree", "somewhat disagree", "somewhat agree", "strongly agree"). A criterion of IGD is met, if at least one of the items has been rated with 3 ("strongly agree"). The instrument thus allows a tentative diagnosis of IGD, if 5 or more criteria are met and a diagnosis or subthreshold IGD if 2-4 criteria are met. Additionally, the CSAS assesses average time spent online per day and provides grade- and age-specific norms for the total score. The instrument shows a high internal consistency reliability coefficient (Cronbach's $\alpha = .94$) and a high face validity due to its proximity to the diagnostic criteria of IGD [38]. As PROTECT is designed as a preventive intervention for IUD (including both gaming and non-gaming internet activities), we adapted the CSAS items accordingly (e.g. item 1: "Even when I am not gaming/ online, I think about online gaming/ the internet" for preoccupation) with permission by the publisher.

3.5.4. Secondary Outcomes

Depressive symptoms are assessed using the German Depression Inventory for Children and Adolescents [39,40]. The instrument contains severity estimations of depressive disorders according to the DSM-5 criteria [3].

The Strength and Difficulties Questionnaire [41] was used to assess behavior problems and strengths. It includes 5 scales ((1) emotional problems, (2) behavior problems, (3) hyperactivity/ attention deficits, (4) interpersonal problems with peers and (5) prosocial behavior) and can be used for epidemiological research and as an indicator for emotional, oppositional, antisocial and attention deficit/ hyperactivity disorder.

The German version of the Social Interaction Anxiety Scale [42,43] is used to assess anxiety in social interactions as an indicator for social anxiety disorders.

Performance anxiety and school anxiety are assessed with the 7th scale of the German adaption of the Fear Survey Schedule for Children – Revised [44,45].

Emotion regulation is measured using the German Questionnaire for Assessment of Emotion Regulation in Children and Adolescents [46]. The questionnaire includes a measure of functional and dysfunctional emotion regulation strategies for the negative emotions fear, sadness and anger.

Procrastination is assessed with the German Questionnaire for Procrastination [47].

For ratings of social competent behavior and academic motivation, we use the German Student Assessment List for Social and Learning Behavior [48]. Self-efficacy is rated on the German Self-Efficacy Scale [49].

The WHO-5 Well-Being Index [50] is a 5 item questionnaire first published in 1998 by the World Health Organization. It assesses subjective psychological well-being on a scale from 0 (worst imaginable well-being) to 100 (best imaginable well-being). The WHO-5 questionnaire as indicator for health related quality of life allows calculating the

quality adjusted life years (QALY), an important index in health economics to determine the cost-utility (CUA) of an intervention. The assessment schedule is presented in Table 2.

Table 2. *Assessment Schedule.*

Measure	T1<br>Baseline	T2<br>1-month-follow-up	T3<br>4-months-follow-up	T4<br>12-months-follow-up
Sociodemographic Variables	x	-	-	-
School Performance / Absence	x	x	x	x
Internet Use Behavior	x	x	x	x
CIUS	x	-	-	-
CSAS	x	x	x	x
DIKJ	x	x	x	x
FEEL-KJ	x	x	x	x
APROF	x	x	x	x
SDQ	x	x	x	x
PHOKI Performance Anxiety	x	x	x	x
SIAS	x	x	x	x
SSL	x	x	x	x
SWE	x	x	x	x
WHO-5	-	-	-	x
Clinical Interview	-	-	-	x
Evaluation (Intervention Group)	x	x	x	x

3.5.5. Evaluation and Feedback

To assess acceptance and satisfaction with the intervention, we developed a feedback-questionnaire. Participants are encouraged to evaluate the trainers, the working atmosphere, the intervention materials, everyday applicability of exercises, identification

with the protagonists of the rational-emotive stories and subjective learning success. Moreover, participants are asked to rate subjective effectiveness of specific interventions (i.e., psychoeducation, cognitive restructuring, behavior modification and emotion regulation), subjective effectiveness of the length of the intervention (dose-response) and subjective effectiveness of the group size on a 5-point Likert scale ("not at all right", "rather not right", "rather right", "exactly right", "no statement"). The feedback items can be allocated on 7 scales (satisfaction, psycho-education, cognitive restructuring, behavior modification, emotion regulation, everyday applicability, identification). Furthermore, participants are asked to indicate their favorite module, key features of the intervention which they found most useful, and to make proposals for improvement. Finally, they are asked to decide whether they would recommend PROTECT to a friend.

3.6. Statistical analyses

The clinical interview allows determining the incidence rate of IUD as well as the time point of illness onset (change point). We will use a hierarchical analysis plan and include methods to control for Type I errors. Since incidence rate and time to incidence were selected as main outcomes, survival analyses are applied to determine efficacy. A Kaplan-Meier approach with Logrank-Test is used as statistical method to compare survival curves between the two groups [51]. Cox-Regression Models are used to identify risk factors and moderators. Changes in prevalence rates and symptom courses (secondary outcomes) are analyzed using Hierarchical Linear Modelling [51].

4. Discussion

The goal of this 2-armed randomized controlled trial is to evaluate the preventive efficacy of a 4-week school-based preventive intervention in adolescents with high-risk for IUD.

It is hypothesized that the intervention decreases incidence rates of IUD in the intervention group trained with PROTECT as compared to the control group and reduces associated psychological problems such as symptoms of IUD, comorbid psychopathology and negative school-related outcomes.

PROTECT follows the APA-guidelines for psychological prevention [52] and is designed as an indicated prevention [53]. It is theory- and evidence-based (guideline 1) and is adapted to various social and cultural backgrounds and thus, accounts for minorities (guideline 2). The intervention addresses both risk-reduction and strength-promotion (guideline 3) by aiming at a reduction of incidence rates and risk factors of IUD and by promoting students' problem solving, cognitive restructuring and emotion regulation skills. It considers current research and epidemiology (guideline 4) and ethical standards such as professional secrecy (guideline 5). Moreover, PROTECT aims at reducing social inequalities by targeting underserved areas (rural areas) and underserved school types associated with lower educational achievement (guideline 6). To account for the quality of the intervention, we implemented a professional training and supervision system for key persons who deliver the preventive intervention, as described in the procedure section (guideline 7). PROTECT is designed as a systemic intervention (guideline 8) at the school-level and implemented in cooperation with policy makers (guideline 9). School-based interventions have been proven to be effective and feasible in other domains [17,54–56]. Especially in the prevention of IUD, school-based approaches seem particularly promising as motivation for treatment is typically low in this target group.

The cognitive behavioral model of IUD [57] defines maladaptive cognitions about the self and the world as well as cognitive distortions such as self-doubt or low self-efficacy as proximal causes for IUD. Following this approach, PROTECT includes cognitive restructuring as a key therapeutic component. Other treatment approaches [58–

61] are equally based on this theoretic framework and have proven to be effective. Furthermore, PROTECT aims at an improvement of problem solving and emotion regulation skills. According to the escape from self-model [62,63], behavioral problems such as IUD result from the discrepancy between the ideal and the real self, which leads to a tendency to escape into virtual reality. The model of compensatory internet use [64] defines IUD as an unconscious effort to compensate for low self-esteem and difficulties in social interactions. Models of behavioral addiction presume that IUD represents a maladaptive, avoidant coping strategy for stressful events [22,65]. In line with various theoretical frameworks, PROTECT uses an interactive approach to promote coping mechanisms as more functional alternatives to excessive internet use by training problem solving skills and emotion regulation strategies. Thus, it exceeds didactic, knowledge-based education approaches, which were only partially effective in previous prevention programs [66].

Other preventive interventions, such as the P.A.T.H.S. project [67] have proven to be effective in reducing time on the internet, severity of internet addiction [66] and in improving student’s self-control of internet use [56,67]. As IUD is a much more common problem in Asian countries, preventive approaches originate from this part of the world, especially from Korea [66] and Hong Kong [67]. In addition to our project, one promising school-based preventive intervention in Germany is currently being implemented and evaluated [68].

As IUD, similar to other addictive disorders, has a possibly high relapse rate, there is an unmet need for preventive interventions addressing distal risk factors of IUD that are also causal for other behavioral problems, instead of only dealing with excessive use of the internet itself [66]. PROTECT targets such distal risk factors, as for instance lack of motivation, performance anxiety, procrastination, and social isolation.

Furthermore, [66] claims that targeting young age groups is crucial in the prevention of IUD. PROTECT is applicable for adolescents from 12 years of age onwards, as the prevalence of IUD is highest in this age group [69]. Although [66] proposes the implementation of long-term interventions, evidence suggests no systematical differences in efficacy between short and long-term behavioral preventive programs and higher dose-response relationships for short interventions [18,20]. Considering the limited time resources in educational settings, short interventions are the preferable option for school-based approaches. To evaluate the efficacy of preventive interventions, more empirical research is needed [66]. There is a lack of high-quality evaluation studies in the field of prevention and treatment of IUD [59,70–72].

In addition to examining the efficacy of PROTECT, we hope to contribute to other open research questions in the field of IUD. The diagnostic criteria of IGD in the research appendix (section 3) of the DSM-5 [3] are only preliminary and are an issue of debate in current research [6,73–75]. In line with Strittmatter and colleagues [6] it is assumable that the restriction of the definition to internet-related gaming is a too narrow approach and that non-gaming internet activities can lead to similar levels of impairment. More empirical data is needed to conclude if an extension of the diagnosis to non-gaming activities is clinically relevant. Therefore, we extended the assessment of the IGD-criteria in the clinical interview and the CSAS [38] for both gaming and non-gaming related internet activities. Furthermore, our study contributes to a better understanding of the phenomenology of subtypes, etiology, risk factors, epidemiology and comorbid psychopathology of IUD. It does not only evaluate the overall efficacy of the preventive intervention on the incidence of IUD, but also differential effects of specific intervention techniques (i.e. cognitive restructuring, problem solving and emotion regulation) on relevant psychological outcomes.

In the process of designing our study, we faced some limitations and difficulties. The main purpose of this preventive intervention is that fewer people develop an IUD. Thus, IUD incidence rates are considered as key outcome criterion. However, in unselected populations, IUD affects only a minority and absolute risk is small. Although the intervention might potentially achieve large effect sizes in relative terms (relative risk reduction), absolute risk reduction is small. We assume that risk may be higher in specific target groups; however, we still have little knowledge about risk factors and only vague speculations about timing and processes. Thus, we have to accept numerous uncertainties in our sample size calculation. Prevalence data of IUD are inconsistent, ranging from 0.8% to 26.7% [2]. Among adolescents, prevalence rates from 1.2 % [4,5] to 6.0% [8] or even 13.3% for subthreshold pathology [9] are reported.

In our design, we assume that 14 % of adolescents are identified as at-risk for IUD. We presume that 36 % of those high-risk adolescents in the control group and 24 % in the intervention group will develop an IUD over the 12-months follow-up period resulting in a population incidence rate of 5% vs. 3.3%. Because of lacking empirical data, we estimate the risk of illness onset within 12 months (incidence rate) on the basis of prevalence data. To assure a power of $\beta = .80$, a total sample size of 340 participants (170 per group) is needed. Only slight changes in the prevalence of at-risk adolescents or in the incidence of IUD in the control group can lead to a significant reduction of power when sample size remains constant. Another problem we face is the difficulty in estimating dropout rates. We assume a dropout-rate of 25 % over the 12-months study period.

To conclude, PROTECT is a promising preventive intervention that aims to reduce the incidence of IUD in adolescent populations. If the efficacy of PROTECT can be established, the program needs to be evaluated after large-scale implementation in

routine care (effectiveness). Further developments of the program should extend its applicability to younger age groups in primary schools or to students with learning difficulties.

References

[1] T.R. Insel, Translating scientific opportunity into public health impact: a strategic plan for research on mental illness, Archives of general psychiatry 66 (2) (2009) 128–133.

[2] D.J. Kuss, M.D. Griffiths, L. Karila, J. Billieux, Internet addiction: a systematic review of epidemiological research for the last decade, Current pharmaceutical design 20 (25) (2014) 4026–4052.

[3] American Psychiatric Association, Diagnostic and Statistical Manual of Mental Disorders, Fifth Edition (DSM-5), American Psychiatric Association, Arlington, 2013.

[4] F. Rehbein, S. Kliem, D. Baier, T. Mößle, N.M. Petry, Prevalence of Internet gaming disorder in German adolescents: diagnostic contribution of the nine DSM-5 criteria in a state-wide representative sample, Addiction 110 (5) (2015) 842–851.

[5] F. Rehbein, M. Kleimann, T. Mößle, Prevalence and risk factors of video game dependency in adolescence: results of a German nationwide survey, Cyberpsychol Behav Soc Netw 13 (3) (2010) 269–277.

[6] E. Strittmatter, M. Kaess, P. Parzer, G. Fischer, V. Carli, C.W. Hoven, C. Wasserman, M. Sarchiapone, T. Durkee, A. Apter, J. Bobes, R. Brunner, D. Cosman, M. Sisask, P. Varnik, D. Wasserman, Pathological Internet use among

adolescents: Comparing gamers and non-gamers, Psychiatry research 228 (1) (2015) 128–135.

[7] E. Strittmatter, M. Kaess, P. Parzer, G. Fischer, V. Carli, C.W. Hoven, C. Wasserman, M. Sarchiapone, T. Durkee, A. Apter, J. Bobes, R. Brunner, D. Cosman, M. Sisask, P. Värnik, D. Wasserman, Pathological Internet use among adolescents_ Comparing gamers and non-gamers.

[8] C. Cheng, A.Y.-l. Li, Internet addiction prevalence and quality of (real) life: a meta-analysis of 31 nations across seven world regions, Cyberpsychology, behavior and social networking 17 (12) (2014) 755–760.

[9] H.J. Rumpf, C. Meyer, U. John, Prävalenz der Internetabhängigkeit (PINTA), Berlin, 2011.

[10] R.A. Mentzoni, G.S. Brunborg, H. Molde, H. Myrseth, K.J.M. Skouveroe, J. Hetland, S. Pallesen, Problematic video game use: estimated prevalence and associations with mental and physical health, Cyberpsychology, behavior and social networking 14 (10) (2011) 591–596.

[11] M.E. Beutel, C. Hoch, K. Wölfling, K.W. Müller, Clinical characteristics of computer game and internet addiction in persons seeking treatment in an outpatient clinic for computer game addiction, Zeitschrift fur Psychosomatische Medizin und Psychotherapie 57 (1) (2011) 77–90.

[12] F. Rehbein, M. Kleimann, T. Mößle, Computerspielabhängigkeit im Kindes- und Jugendalter: Empirische Befunde zu Ursachen, Diagnostik und Komorbiditäten unter besonderer Berücksichtigung spielimmanenter Abhängigkeitsmerkmale, Hannover, 2009.

[13] S. Achab, M. Nicolier, F. Mauny, J. Monnin, B. Trojak, P. Vandel, D. Sechter, P. Gorwood, E. Haffen, Massively multiplayer online role-playing games: comparing

characteristics of addict vs non-addict online recruited gamers in a French adult population, BMC psychiatry 11 (2011) 144.

[14] V. Carli, T. Durkee, D. Wasserman, G. Hadlaczky, R. Despalins, E. Kramarz, C. Wasserman, M. Sarchiapone, C.W. Hoven, R. Brunner, M. Kaess, The association between pathological internet use and comorbid psychopathology: a systematic review, Psychopathology 46 (1) (2013) 1–13.

[15] D.A. Gentile, H. Choo, A. Liau, T. Sim, D. Li, D. Fung, A. Khoo, Pathological video game use among youths: a two-year longitudinal study, Pediatrics 127 (2) (2011) e319-e329.

[16] Die Drogenbeauftragte der Bundesregierung, Drogen- und Suchtbericht 2016, 2016.

[17] K. Lindenberg, H. Kordy, Wirksamkeit eines gestuften, internetvermittelten Ansatzes zur Prävention von Essstörungen bei Schülern der 7. bis 10. Klasse. [Efficacy of an internet-delivered tiered strategy for eating disorder prevention in high school students]., Kindheit und Entwicklung 24 (1) (2015) 55–63.

[18] J. Junge-Hoffmeister, Prävention Psychischer Störungen, in: S. Schneider, J. Margraf (Eds.), Lehrbuch der Verhaltenstherapie: Band 3: Störungen im Kindes- und Jugendalter, Springer Medizin Verlag, Heidelberg, 2009, pp. 901–922.

[19] K. Lindenberg, Gestufte Prävention von Essstörungen mit YoungEs[s]prit: Wirksamkeit eines Internet-vermittelten Präventionsprogramms bei Schülern. Dissertation, Heidelberg, 2012.

[20] J.L. Horowitz, J. Garber, The prevention of depressive symptoms in children and adolescents: A meta-analytic review, J Consult Clin Psychol 74 (3) (2006) 401–415.

[21] G.-J. Meerkerk, R. Van Den Eijnden, A. Vermulst, H. Garretsen, The Compulsive Internet Use Scale (CIUS): Some psychometric properties, CyberPsychology & Behavior 12 (1) (2009) 1–6.

[22] K. Wölfling, C. Jo, I. Bengesser, M.E. Beutel, K.W. Müller, Computerspiel- und Internetsucht: Ein kognitiv-behaviorales Behandlungsmanual, Kohlhammer, Stuttgart, 2013.

[23] K. Lindenberg, S. Schoenmaekers, S. Szász-Janocha, U. Wehrmann and E. Vonderlin (2017). Verbesserung der Zugangs- und Versorgungswege für junge Menschen mit Pathologischem Internetgebrauch: Ansätze zur Prävention, Frühintervention und integrierten Versorgung. *SuchtAktuell* **1**, 2017, 5-10..

[24] H.H. Stavemann, KVT-Praxis: Strategien und Leitfäden für die kognitive Verhaltenstherapie, Beltz, Weinheim, 2005.

[25] H.H. Stavemann, Im Gefühlsdschungel: Emotionale Krisen verstehen und bewältigen, 2nd ed., Beltz, Weinheim, Basel, 2010.

[26] J.G. Allen, P. Fonagy (Eds.), Mentalisierungsgestützte Therapie: Das MBT-Handbuch - Konzepte und Praxis, Klett-Cotta, Stuttgart, 2009.

[27] V. Waters, D. Schwartz, Fritzchen Flunder und Nora Nachtigall: Sechs rational-emotive Geschichten zum Nachdenken für Kinder, 1st ed., Huber, Bern, 2003.

[28] A.A. Schlarb (Ed.), Praxisbuch KVT mit Kindern und Jugendlichen: Störungsspezifische Strategien und Leitfäden, 1st ed., Beltz, Weinheim, 2012.

[29] H.H. Stavemann, Sokratische Gesprächsführung in Therapie und Beratung: Eine Anleitung für Psychotherapeuten, Berater und Seelsorger, 1st ed., Beltz PVU, Weinheim, 2002.

[30] A. Beyer, Stressbewältigung im Jugendalter: Ein Trainingsprogramm, Hogrefe, Göttingen, 2006.

[31] M. Cierpka, Faustlos - wie Kinder Konflikte gewaltfrei lösen lernen, 8th ed., Herder, Freiburg im Breisgau, 2011.

[32] G. Lauth, K. Mackowiak, Kognitive Verfahren, in: S. Schneider, J. Margraf (Eds.), Lehrbuch der Verhaltenstherapie: Band 3: Störungen im Kindes- und Jugendalter, Springer Medizin Verlag, Heidelberg, 2009, pp. 221–232.

[33] P. Heppner, E. Hillerbrand, Problem-Solving-Training: Implications for Remedial and Preventive Training, in: C.R. Snyder (Ed.), Handbook of social and clinical psychology: The health perspective, Pergamon Press, New York, 1999, pp. 681–698.

[34] U. Petermann, J. Pätel, Entspannungsverfahren, in: S. Schneider, J. Margraf (Eds.), Lehrbuch der Verhaltenstherapie: Band 3: Störungen im Kindes- und Jugendalter, Springer Medizin Verlag, Heidelberg, 2009, pp. 243–254.

[35] H. Saß, Diagnostische Kriterien des diagnostischen und statistischen Manuals psychischer Störungen DSM-IV-TR, Hogrefe, Göttingen, 2003.

[36] G. Bischof, A. Bischof, Meyer, C., John, U., H.-J. Rumpf, Prävalenz der Internetabhängigkeit – Diagnostik und Risikoprofile (PINTA-DIARI), Lübeck, 2013.

[37] R. Tao, X. Huang, J. Wang, H. Zhang, Y. Zhang, M. Li, Proposed diagnostic criteria for internet addiction, Addiction 105 (3) (2010) 556–564.

[38] F. Rehbein, D. Baier, M. Kleimann, T. Mößle, Computerspielabhängigkeitsskala (CSAS): ein Verfahren zur Erfassung der Internet Gaming Disorder nach DSM-5, Hogrefe, Göttingen, Bern, Wien, 2015.

[39] J. Stiensmeier-Pelster, M. Schürmann, K. Duda, Depressions-Inventar für Kinder und Jugendliche (DIKJ): Handanweisung, Verl. für Psychologie Hogrefe, Göttingen, 1989.

[40] J. Stiensmeier-Pelster, M. Braune-Krickau, M. Schürmann, K. Duda, DIKJ: Manual, 3rd ed., Hogrefe, Göttingen, 2014.

[41] R. Goodman, H. Meltzer, V. Bailey, The Strengths and Difficulties Questionnaire: a pilot study on the validity of the self-report version, International Review of Psychiatry 15 (1-2) (2003) 173–177.

[42] R.P. Mattick, J. Clarke, Development and validation of measures of social phobia scrutiny fear and social interaction anxiety, Behaviour Research and Therapy 36 (4) (1998) 455–470.

[43] U. Stangier, T. Heidenreich, A. Berardi, U. Golbs, J. Hoyer, Die Erfassung sozialer Phobie durch die Social Interaction Anxiety Scale (SIAS) und die Social Phobia Scale (SPS), Zeitschrift für Klinische Psychologie und Psychotherapie 28 (1) (1999) 28–36.

[44] M. Döpfner, M. Schnabel, H. Goletz, T. Ollendick, Phobiefragebogen für Kinder und Jugendliche: PHOKI, Hogrefe, Göttingen, 2006.

[45] P. Muris, T.H. Ollendick, The assessment of contemporary fears in adolescents using a modified version of the Fear Survey Schedule for Children-Revised, Journal of anxiety disorders 16 (6) (2002) 567–584.

[46] A. Grob, C. Smolenski, Fragebogen zur Erhebung der Emotionsregulation bei Kindern und Jugendlichen: FEEL-KJ; Manual, 2nd ed., Huber, Bern, 2011.

[47] A. Höcker, M. Engberding, F. Rist, Prokrastination: ein Manual zur Behandlung des pathologischen Aufschiebens, Hogrefe Verlag, Göttingen, 2013.

[48] U. Petermann, F. Petermann, A. Lohbeck, Schülereinschätzliste für Sozial- und Lernverhalten: SSL, Hogrefe, Göttingen, 2014.

[49] R. Schwarzer, M. Jerusalem, Skalen zur Erfassung von Lehrer- und Schülermerkmalen: Dokumentation der psychometrischen Verfahren im Rahmen

der Wissenschaftlichen Begleitung des Modellversuchs Selbstwirksame Schulen, 1999.

[50] C.W. Topp, S.D. Ostergaard, S. Sondergaard, P. Bech, The WHO-5 Well-Being Index: a systematic review of the literature, Psychotherapy and psychosomatics 84 (3) (2015) 167–176.

[51] J.D. Singer, J.B. Willett, Applied longitudinal data analysis: Modeling change and event occurrence, Oxford University Press, New York, 2003.

[52] American Psychological Association, Guidelines for prevention in psychology, The American psychologist 69 (3) (2014) 285–296.

[53] R.S. Gordon, An operational classification of disease prevention, Public Health Reports 98 (2) (1983) 107–109.

[54] E. Skryabina, G. Taylor, P. Stallard, Effect of a universal anxiety prevention programme (FRIENDS) on children's academic performance: results from a randomised controlled trial, Journal of child psychology and psychiatry, and allied disciplines (2016).

[55] S.M. Wilksch, School-based eating disorder prevention: a pilot effectiveness trial of teacher-delivered Media Smart, Early intervention in psychiatry 9 (1) (2015) 21–28.

[56] D.T.L. Shek, L. Yu, Prevention of adolescent problem behavior: longitudinal impact of the Project P.A.T.H.S. in Hong Kong, TheScientificWorldJournal 11 (2011) 546–567.

[57] R.A. Davis, A cognitive-behavioral model of pathological Internet use, Computers in Human Behavior 17 (2) (2001) 187–195.

[58] Y.-s. Du, W. Jiang, A. Vance, Longer term effect of randomized, controlled group cognitive behavioural therapy for Internet addiction in adolescent students in

Shanghai, The Australian and New Zealand journal of psychiatry 44 (2) (2010) 129–134.

[59] D.L. King, P.H. Delfabbro, M.D. Griffiths, M. Gradisar, Cognitive-behavioral approaches to outpatient treatment of internet addiction in children and adolescents, Journal of clinical psychology 68 (11) (2012) 1185–1195.

[60] K.S. Young, Treatment outcomes using CBT-IA with Internet-addicted patients, Journal of behavioral addictions 2 (4) (2013) 209–215.

[61] K.S. Young, CBT-IA: The First Treatment Model for Internet Addiction, J Cogn Psychother 25 (4) (2011) 304–312.

[62] J.-H. Kwon, C.-S. Chung, J. Lee, The effects of escape from self and interpersonal relationship on the pathological use of Internet games, Community mental health journal 47 (1) (2011) 113–121.

[63] R.F. Baumeister, Suicide as escape from self, Psychological review 97 (1) (1990) 90–113.

[64] D. Kardefelt-Winther, A conceptual and methodological critique of internet addiction research: Towards a model of compensatory internet use, Computers in Human Behavior 31 (2014) 351–354.

[65] S.M. Grüsser, C.N. Thalemann, Verhaltenssucht: Diagnostik, Therapie, Forschung, 1st ed., Huber, Bern, 2006.

[66] J.-H. Kwon, Toward the prevention of adolescent Internet addiction., in: K.S. Young, C.N. de Abreu (Eds.), Internet addiction: A handbook and guide to evaluation and treatment, John Wiley & Sons, Hoboken, NJ, 2011, pp. 223–243.

[67] D. Busiol, T.Y. Lee, Prevention of Internet Addiction: The P.A.T.H.S. Program, in: T.Y. Lee, D.T. Shek, R.C.F. Sun (Eds.), Student Well-Being in Chinese Adolescents in Hong Kong, Springer Singapore, Singapore, 2015, pp. 185–193.

[68] M. Dreier, K. Wölfling, M.E. Beutel, K.W. Müller, Prevention of Internet Addiction, Pädiatrie & Pädologie 50 (5) (2015) 200–205.

[69] J. Xu, L.-x. Shen, C.-h. Yan, H. Hu, F. Yang, L. Wang, S.R. Kotha, L.-n. Zhang, X.-p. Liao, J. Zhang, F.-x. Ouyang, J.-s. Zhang, X.-m. Shen, Personal characteristics related to the risk of adolescent internet addiction: a survey in Shanghai, China, BMC Public Health 12 (1) (2012) 1106.

[70] D.L. King, P.H. Delfabbro, Internet gaming disorder treatment: a review of definitions of diagnosis and treatment outcome, Journal of clinical psychology 70 (10) (2014) 942–955.

[71] D.L. King, P.H. Delfabbro, M.D. Griffiths, M. Gradisar, Assessing clinical trials of Internet addiction treatment: a systematic review and CONSORT evaluation, Clin Psychol Rev 31 (7) (2011) 1110–1116.

[72] D.J. Kuss, O. Lopez-Fernandez, Internet addiction and problematic Internet use: A systematic review of clinical research, World journal of psychiatry 6 (1) (2016) 143–176.

[73] M. Lehenbauer-Baum, A. Klaps, Z. Kovacovsky, K. Witzmann, R. Zahlbruckner, B.U. Stetina, Addiction and Engagement: An Explorative Study Toward Classification Criteria for Internet Gaming Disorder, Cyberpsychology, behavior and social networking 18 (6) (2015) 343–349.

[74] M. Lehenbauer-Baum, M. Fohringer, Towards classification criteria for internet gaming disorder: Debunking differences between addiction and high engagement in a German sample of World of Warcraft players, Computers in Human Behavior 45 (2015) 345–351.

[75] A. Musetti, R. Cattivelli, M. Giacobbi, P. Zuglian, M. Ceccarini, F. Capelli, G. Pietrabissa, G. Castelnuovo, Challenges in Internet Addiction Disorder: Is a Diagnosis Feasible or Not?, Frontiers in psychology 7 (2016) 842.
