# Supplementary material for: Effectiveness of Cognitive Behavioral Therapy–Based Intervention in Preventing Gaming Disorder and Unspecified Internet Use Disorder in Adolescents: A Cluster Randomized Clinical Trial
Source: JAMA Netw Open. 2022 Feb 18;5(2):e2148995. doi: 10.1001/jamanetworkopen.2021.48995 (PMC8857686; doi:10.1001/jamanetworkopen.2021.48995)
Supplement: Supplement 3. — Data Sharing Statement [file jamanetwopen-e2148995-s003.pdf]

Data Sharing Statement

Lindenberg. Effectiveness of Cognitive Behavioral Therapy-Based Intervention in Preventing Gaming Disorder and Unspecified Internet Use Disorder in Adolescents. *JAMA Netw Open*. Published February 18, 2022. doi:10.1001/jamanetworkopen.2021.48995

Data
Data available: No

Additional Information
Explanation for why data not available: on request via email: lindenberg@psych.uni-frankfurt.de
